# Supplementary material for: Harmonic amide bond density as a game-changer for deciphering the crosslinking puzzle of polyamide
Source: Nat Commun. 2024 Feb 20;15:1539. doi: 10.1038/s41467-024-45918-4 (PMC10879148; doi:10.1038/s41467-024-45918-4)
Supplement: Supplementary file 1 — Supplementary Information [file 41467_2024_45918_MOESM1_ESM.pdf]

Supplementary Information

Harmonic Amide Bond Density as a Game-Changer for Deciphering the Crosslinking Puzzle of Polyamide

*Yu-Ren Xue [1,2]†, Chang Liu [1,2]†, Zhao-Yu Ma [1,2], Cheng-Ye Zhu [1,2], Jian Wu [3], Hong-Qing Liang [1,2] *, Hao-Cheng Yang [1,2], Chao Zhang [1,2], Zhi-Kang Xu [1,2] **

[1] Key Lab of Adsorption and Separation Materials & Technologies of Zhejiang Province, MOE Engineering Research Center of Membrane and Water Treatment, Department of Polymer Science and Engineering, Zhejiang University, Hangzhou 310027, China

[2] The "Belt and Road" Sino-Portugal Joint Lab on Advanced Materials, International Research Center for X Polymers, Zhejiang University, Hangzhou 310058, China

[3] Department of Chemistry, Zhejiang University, Hangzhou 310058, China

Corresponding Author: Dr. Hong-Qing Liang (liang.hongqing@zju.edu.cn), Prof. Dr. Zhi-Kang Xu (xuzk@zju.edu.cn).

This PDF includes:

Supplementary Text

Supplementary Figures 1 to 22

Supplementary Formula 2-1 to 2-13, 3-1 to 3-4, 4-1 to 4-9, 6-1 to 6-13

Supplementary Tables 1 to 16

Supplementary References and Notes

Contents

1. Molecular dynamics (MD) simulations of MPD-TMC polyamide 1

2. HABD for desalination polyamide membranes 2

2.1. Segmentation of polyamide 2

2.2. Fractional free volume of polyamide 2

2.3. Calculation of HABD in MPD-TMC polyamide 3

2.4. Calculation of HABD in PIP-TMC polyamide 5

3. Separation performance of desalination membranes 11

3.1. Performance of desalination membranes 11

3.2. Rejection, permeability of NaCl and water permeance in RO and DNC 11

3.3. Rejection, permeability of NaCl and water permeance in RO and HABD 18

3.4. Correlation between NaCl permeability in RO with HABD or DNC 20

3.5. Rejection, permeability of Na_2SO_4 and water permeance in NF and DNC 20

3.6. Rejection, permeability of Na_2SO_4 and water permeance in NF and HABD 26

3.7. Correlation between Na_2SO_4 permeability in NF and DNC or HABD 28

3.8. Water permeability in RO 29

3.9. Water permeability in NF 33

4. HCD and HAD for quantifying terminal groups 39

4.1. Calculation of HCD and HAD in PIP-TMC polyamide 39

4.2. HCD and HAD with ionization of terminal groups 39

4.3. Zeta potential in NF and HCD 40

4.4. Correlation between Zeta potential in NF and HCD 43

5. XPS test conditions 44

5.1. XPS test conditions in reference for obtaining XPS data 44

5.2. Recommended XPS testing conditions 53

6. HABD for new polyamide membranes 55

6.1. TAM-TMC polyamide 58

6.2. TAPE-TMC polyamide 60

6.3. TAPE-BTEC polyamide 61

6.4. PEI-TMC polyamide 63

7. References and notes 65

1. Molecular dynamics (MD) simulations of MPD-TMC polyamide

In the structural simulation of polyamides, simulated crosslinking was employed to construct an all-atom molecular model of polyamide. Deprotonated MPD and TMC with removed chlorine atoms served as the main building blocks. To introduce hydrolysis effects during modeling, some acyl chloride groups in TMC were substituted with carboxyl groups. The complete monomers were constructed with Gaussview and optimized via Gaussian 16 software package [1] at the B3LYP-D3(BJ) [2,3] level of theory with a def2-TZVP basis set [4]. These optimized monomers were then modified into building blocks for further use.

The simulation box, comprising an orthogonal periodic cell with dimensions of 70 × 70 × 70 $Å^3$, was filled with different building blocks using Packmol [5] at a density of 0.4 g cm^{-3}. The crosslinking process was simulated using the Polymatic program package [6] under the polymer consistent force field (PCFF) [7], using RESP2 atomic charges calculated by Multiwfn [8]. This package methodically identified crosslinking chemical bonds by assessing the distance and molecular angles between atoms ready for bonding. Key bonding atoms, specifically amino nitrogen and carbonyl carbon, were provisionally assigned virtual charges of -0.5 and +0.5, respectively. Upon satisfying the bonding criteria, these atoms were connected to form an amide bond, followed by the removal of the virtual charges, and subsequent energy minimization and MD relaxation. After the simulated crosslinking process concluded, amino nitrogen atoms that did not participate in crosslinking were saturated with hydrogen atoms, and uninvolved carbonyl carbon atoms were saturated with hydroxyl groups. The finalized structure then underwent a 21-step simulated annealing procedure using the LAMMPS program, facilitating structural relaxation under diverse temperature and pressure conditions to yield a fully atomistic model of the crosslinked polyamide. The supplementary settings for the simulated crosslinking were adapted from the methodologies of Abbott et al. and our preceding research [6,9].

The fractional free volume within the polyamide networks, considering different hydrolysis sites, were computed using the ZEO++ program with a probe radius of 0.1 Å, based on Voronoi decomposition. The molecular volumes of the structure cells were calculated employing the Multiwfn program, which utilized the Marching Tetrahedron algorithm with an isosurface of 0.002 a.u.[8,10]. All visualizations of the structures were generated using VMD 1.9.4[11].

2. HABD for desalination polyamide membranes

2.1. Segmentation of polyamide

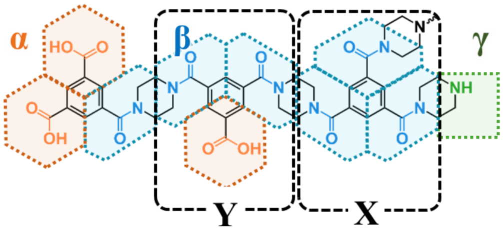

Supplementary Fig. 1. Comparison of harmonic amide bond density (HABD) and degree of network crosslinking (DNC) in analyzing the structures and describing performances of polyamide membranes (with PIP-TMC polyamide as an example).

2.2. Fractional free volume of polyamide

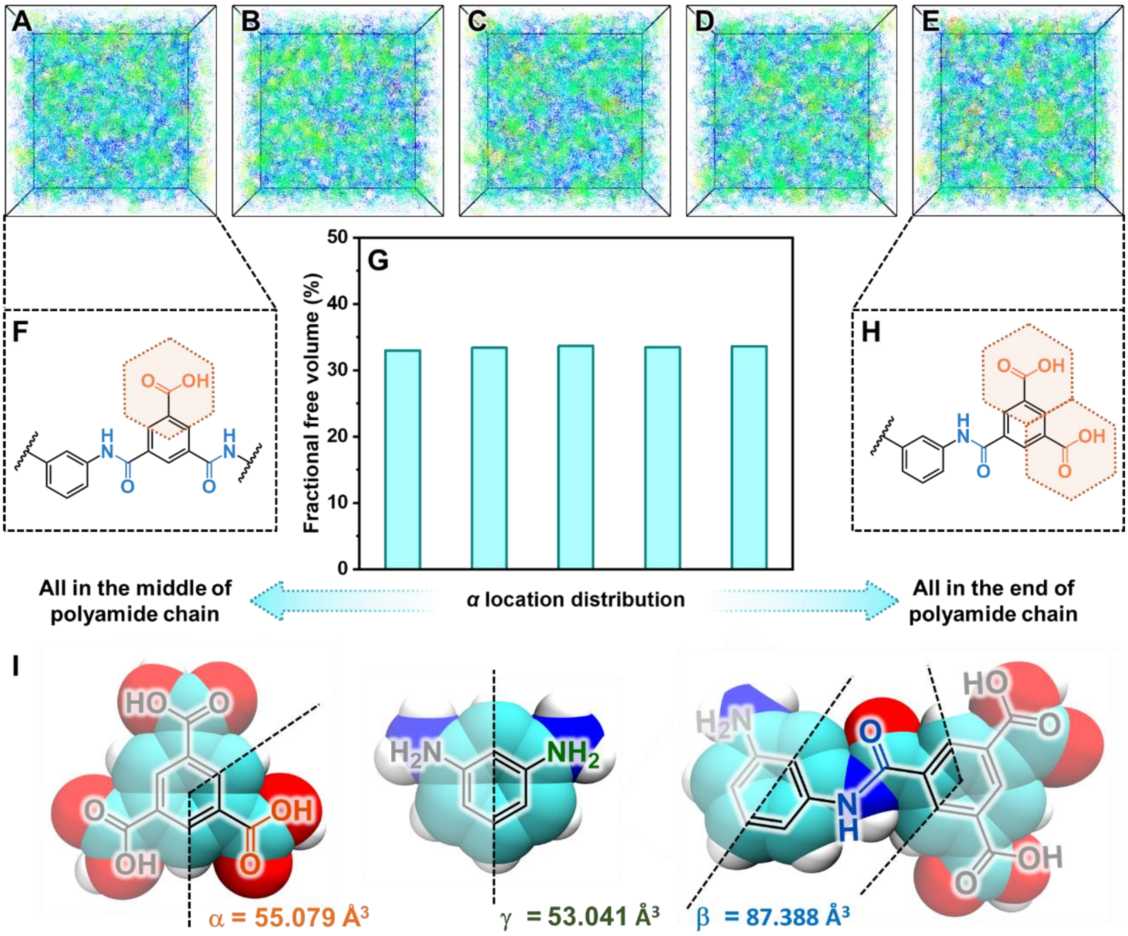

Supplementary Fig. 2. Fractional free volume of five types of polyamides with different structure cell distributions. Almost identical distribution positions of vacancy (The green to yellow dots indicate the size

of the vacancy from small to large) in different polyamide crosslinked networks with 100%(A), 75%(B), 50%(C), 25%(D), 0%(E) of structure cell α located in the middle of polyamide chain respectively. Structural formula of parts of MPD-TMC polyamide when structure cell α located in the middle (F) or in the middle (H) of polyamide chain respectively. Polyamides with different α location distribution own similar fractional free volume (G). Van der Waals volumes of structure cells α, β, and γ in condensed phase are calculated from corresponding molecules using electron density isosurfaces (I).

2.3. Calculation of HABD in MPD-TMC polyamide

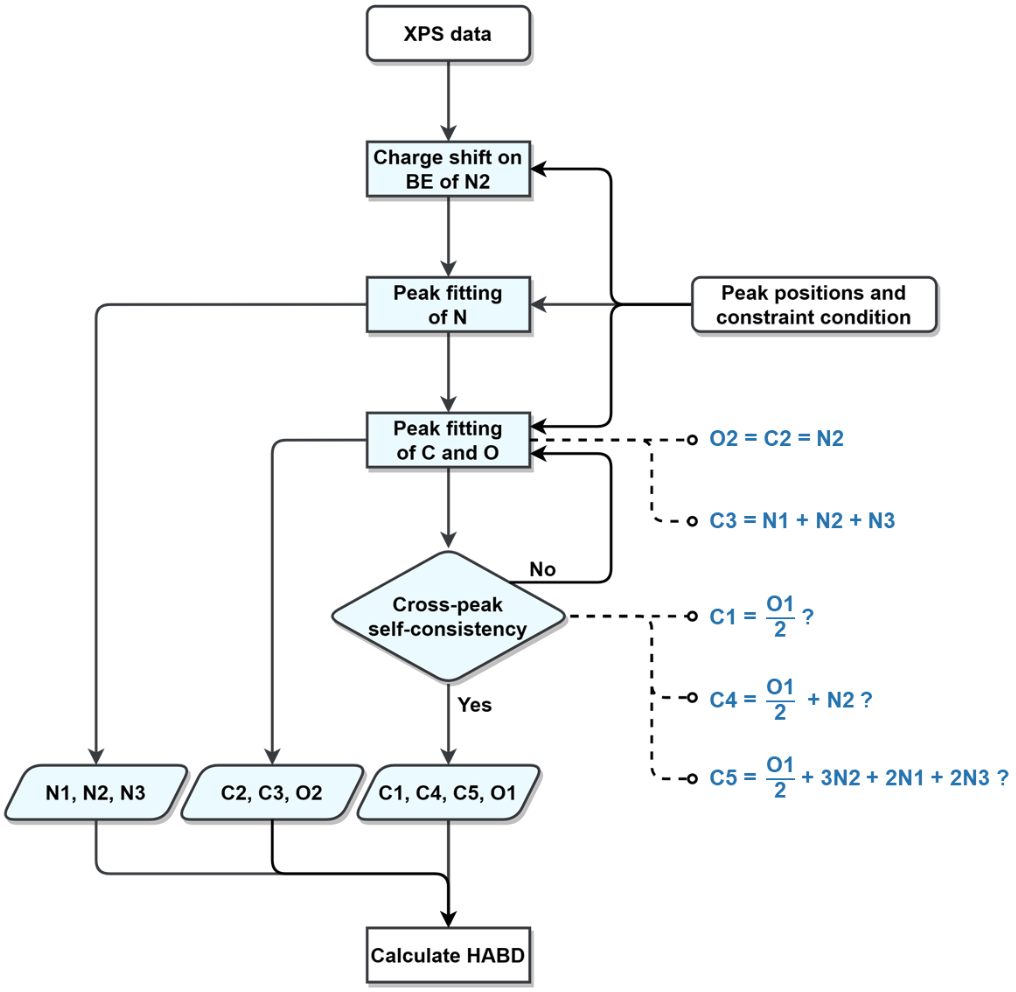

Supplementary Fig. 3. Flowchart of XPS data analysis process of MPD-TMC polyamide.

Supplementary Table 1. Classification and the number of various atoms of α, β, and γ structure cells in MPD-TMC polyamide and recommended peak positions for XPS peak fitting of MPD-TMC polyamide [12-14].

Type	Group	MPD-TMC polyamide			
		XPS peak position	α	β	γ
C1	-C-COOH	288.8 ± 0.1 eV	1	0	0
C2	-C-CONH-	288.3 ± 0.2 eV	0	1	0
C3	-C-NH_2, -CONH-C-,	286.3 ± 0.2 eV	0	1	1
C4	-C-COOH, -C-CONH-	285.7 ± 0.2 eV	1	1	0
C5	-C-H, -C-C, -C=C	284.8 ± 0.2 eV	1	3	2
O1	-COOH	533.1 ± 0.3 eV	2	0	0
O2	-CONH-	531.6 ± 0.4 eV	0	1	0
N1	-NH_2	398.5 ± 0.2 eV	0	0	1
N3	-NH_3^+	401.7 ± 0.2 eV			
N2	-CONH-	400.0 eV	0	1	0

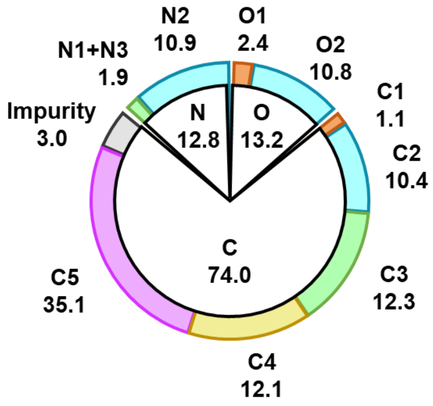

Supplementary Fig. 4. Recommended atom proportion result of N, O, and C of example MPD-TMC polyamide. The polyamide example was synthesized according to the following conditions: [MPD] = 20.0 g L^{-1}, [TMC] = 1.5 g L^{-1}, reaction time = 120 s. XPS test measured through Thermo Scientific K-Alpha+ at Al Kα line (1486.6 eV, 15 mA×15 kV), the vacuum level is 5×10^{-9} mbar, X-ray beam spot is 300 μm, total X-ray irradiation time is 154.1 s (68 s for full spectrum, 28.7 s for C, 30.2 s for O, and 27.2 s for N).

For MPD-TMC polyamide, an amino structure cell γ only contains one N1 or N3, while an amide bond structure cell β only contains one N2:

$$\frac{N1+N3}{N2}=\frac{\gamma}{\beta} \tag{2-1}$$

A carboxyl structure cell α contains two O1, while an amide bond structure cell β only contains one O2:

$$\frac{O1}{O2}=\frac{2\alpha}{\beta} \tag{2-2}$$

HABD equal to:

$$\mathrm{HABD}=\frac{\beta}{\alpha M_\alpha+\beta M_\beta+\gamma M_\gamma}=\frac{1}{\frac{\alpha}{\beta}M_\alpha+M_\beta+\frac{\gamma}{\beta}M_\gamma} \tag{2-3}$$

Therefore:

$$\mathrm{HABD}=\frac{1}{\frac{O1}{2O2}M_\alpha+M_\beta+\frac{N1+N3}{N2}M_\gamma} \tag{2-4}$$

2.4. Calculation of HABD in PIP-TMC polyamide

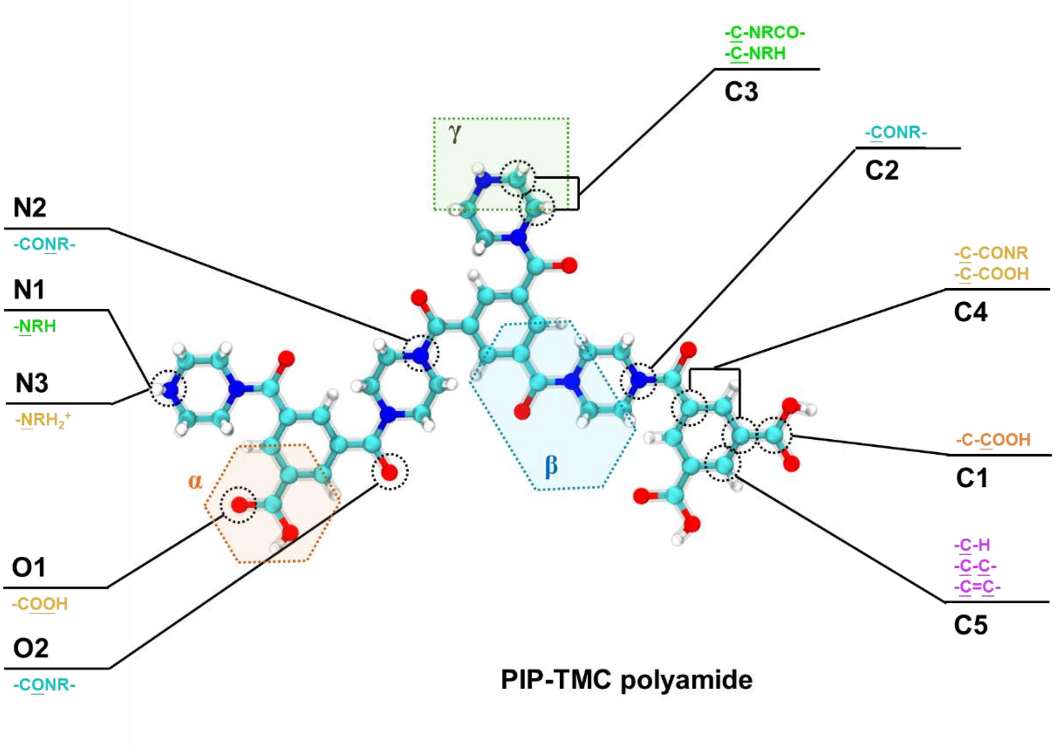

Supplementary Fig. 5. Atomic classification of PIP-TMC polyamide.

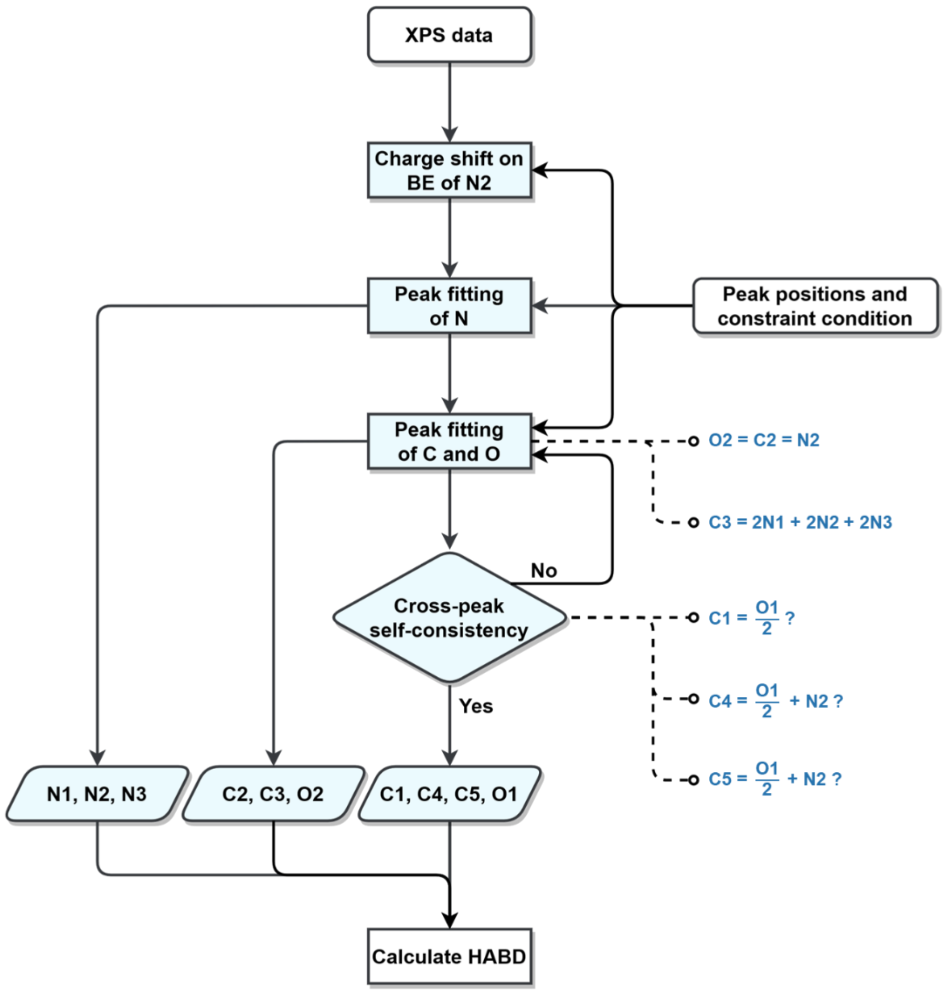

Supplementary Fig. 6. Flowchart of XPS data analysis process of PIP-TMC polyamide.

Supplementary Table 2. Classification and the number of various atoms of α, β, and γ structure cells in PIP-TMC polyamide and recommended peak positions for XPS peak fitting of PIP-TMC polyamide [12-14].

Type	Group	PIP-TMC polyamide			
		XPS peak position	α	β	γ
C1	-C-COOH	288.8 ± 0.1 eV	1	0	0
C2	-C-CONR-	288.3 ± 0.2 eV	0	1	0
C3	-C-NH-, -CONR-C-,	286.3 ± 0.2 eV	0	2	2
C4	-C-COOH, -C-CONR-	285.7 ± 0.2 eV	1	1	0
C5	-C-H, -C-C, -C=C	284.8 ± 0.2 eV	1	1	0
O1	-COOH	533.1 ± 0.3 eV	2	0	0

O2	-CONR-	531.6 ± 0.4 eV	0	1	0
N1	-NH-	398.5 ± 0.2 eV	0	0	1
N3	$-NH_2^+-$	401.7 ± 0.2 eV			
N2	-CONR-	400.0 eV	0	1	0

To accurately determine the values of HABD of PIP-TMC polyamide, here is a matching XPS data processing method and HABD calculation method after the segmentation of PIP-TMC polyamide (Supplementary Fig. 5 and 6). Firstly, we categorize the atoms of polyamide based on the structure cell segmentation (Supplementary Table 2). Nitrogen (N) atoms are divided into amino N atoms (N1, in amino group), amide N atoms (N2, in amide bond), and protonated amino N atoms (N3, in amino group), while Oxygen (O) atoms are categorized into non-amide O atoms (O1) and amide O atoms (O2, in amide bond). Carbon (C) atoms are also divided into carboxyl C atoms (C1, in carboxyl group), amide C atoms (C2, in amide bond), N-related C atoms (C3, connected to the N atom, but not in amide bond), and O-related C atoms (C4, connected to O atom, but not in carboxyl group) and common C atoms (C5).

Subsequently, we utilize the N element (specifically, binding energy of N2) for the charge shift of the full-spectrum. The N peak in polyamide is mainly contributed by the N atom of the amide bond, which minimally affected and interfered by external impurities, making its N2 peak the most easily identifiable. This step is beneficial for subsequent data analysis and peak fitting process, although it does not significantly improve the accuracy of the analysis.

Then, the N peak is divided into three peaks based on the relative peak positions of N1, N2 and N3 as outlined in the relevant research or handbooks (Supplementary Fig. 7A and Table 2). Peak fitting should be carried out under Gaussian–Lorentzian product pseudo-Voigt peak shape, with a Gaussian component comprising 60% ~ 80% of the peak, as Gaussian components are commonly observed in XPS peaks of polymers [15,16]. Throughout this process, attention should be paid to constraining the peak positions of the three N segments within the recommended range while ensuring that their full width at half maxima (FWHM) are similar.

The peak fitting of C and O is carried out with strict adherence to the cross-peak self-consistent relationship,[17] in addition to considering curve shape, peak position, and FWHM.

(Supplementary Fig. 7B and C). The proportion of O2 and C2 can be determined based on the self-consistent relationship between the elements within the amide bond or amino in polyamide. In a single amide bond, there should be one C atom, one O atom, and one N atom, resulting in equal quantities of C2, O2, and N2.

$$C2 = O2 = N2 \tag{2-5}$$

In PIP-TMC, each N atom is connected to two C atoms in piperazine ring, implying that the number of C3 should be equal to:

$$C3 = 2N1 + 2N2 + 2N3 \tag{2-6}$$

The content of C1, C4, C5, and O1, cannot be directly calculated through the peak fitting results of N. However, self-consistent relationships also impose constraints on their cross-peak self-consistency.

In the absence of impurities, both C and O originate from polyamide. A carboxyl group consist of two O1 atoms, one C1 atom and is connected to a C4 atom. Similarly, an amide bond is also connected to a C4 atom, thus:

$$C1 = \frac{O1}{2} \tag{2-7}$$

$$C4 = \frac{O1}{2} + N2 \tag{2-8}$$

According to the division and statistics of our structure cells, we can also obtain statistical relationships:

$$C5 = \frac{O1}{2} + N2 \tag{2-9}$$

The remaining atoms that cannot be separated should be considered as impurities, rather than forcibly attributed to C1, C4, C5 or O1. For example, if the total number of C after peak fitting exceeds the sum of C1-C5 under these constraint conditions, it indicates the presence of impurity C in the XPS test. Similarly, if the total number of C is insufficient to satisfy the Supplementary Formula 2-7 to 2-9 after dividing the peak of O, it indicates that some impurities in O have been separated and attributed to O1.

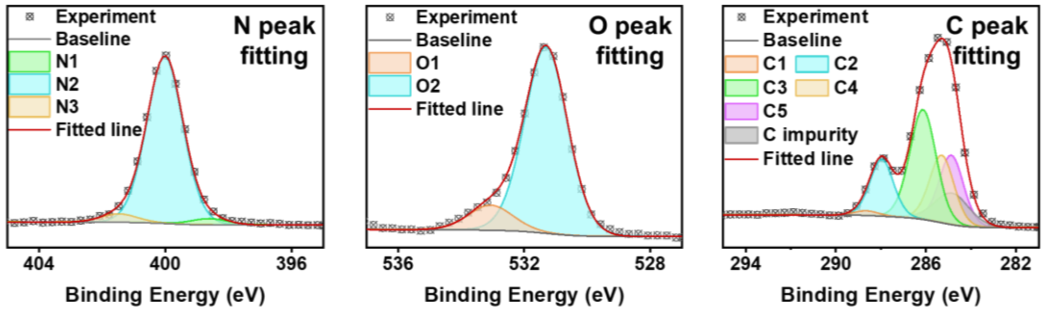

Supplementary Fig. 7. Recommended peak fitting result of N, O, and C of example PIP-TMC polyamide. The polyamide example were synthesized according to the following conditions: [PIP] = 1.0 g L^{-1}, [TMC] = 1.5 g L^{-1}, reaction time = 120 s. XPS test measured through Thermo Scientific K-Alpha+ at Al Kα line (1486.6 eV, 15 mA×15 kV), the vacuum level is 5×10^{-9} mbar, X-ray beam spot is 300 μm, total X-ray irradiation time is 154.1 s (68 s for full spectrum, 28.7 s for C, 30.2 s for O, and 27.2 s for N).

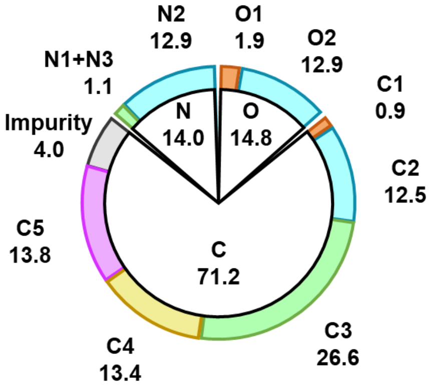

Supplementary Fig. 8. Recommended atom proportion result of N, O, and C of example PIP-TMC polyamide. The polyamide example were synthesized according to the following conditions: [PIP] = 1.0 g L^{-1}, [TMC] = 1.5 g L^{-1}, reaction time = 120 s. XPS test measured through Thermo Scientific K-Alpha+ at Al Kα line (1486.6 eV, 15 mA×15 kV), the vacuum level is 5×10^{-9} mbar, X-ray beam spot is 300 μm, total X-ray irradiation time is 154.1 s (68 s for full spectrum, 28.7 s for C, 30.2 s for O, and 27.2 s for N).

Supplementary Fig. 7 and 8 show the peak fitting results of our self-prepared sample. We use N as the standard for correction. N peak is fully attributed to different N atoms within the polyamide. When the peak conditions and cross-peak self-consistency constraints are met, the

C peak does not exclusively correspond to O1~O2 and C1~C5 atoms of polyamide. The remaining portions are treated as impurities with unspecific peak positions. The quantities of various atoms (Supplementary Fig. 8) must adhere to the self-consistent requirements in Supplementary Formula 2-5 to 2-9, which is a crucial aspect of our method. Once these values of N1, N2, N3, O1, and O2 are obtained, HABD can be calculated by further process:

Similar to MPD-TMC polyamide, for PIP-TMC polyamide, an amino structure cell γ only contains one N1 or N3 as well, while an amide bond structure cell β only contains one N2:

$$\frac{\mathrm{N1+N3}}{\mathrm{N2}}=\frac{\gamma}{\beta} \tag{2-10}$$

A carboxyl structure cell α contains two O1, while an amide bond structure cell β only contains one O2:

$$\frac{\mathrm{O1}}{\mathrm{O2}}=\frac{2\alpha}{\beta} \tag{2-11}$$

HABD equal to:

$$\mathrm{HABD}=\frac{\beta}{\alpha M_\alpha+\beta M_\beta+\gamma M_\gamma}=\frac{1}{\frac{\alpha}{\beta}M_\alpha+M_\beta+\frac{\gamma}{\beta}M_\gamma} \tag{2-12}$$

Therefore:

$$\mathrm{HABD}=\frac{1}{\frac{\mathrm{O1}}{2\mathrm{O2}}M_\alpha+M_\beta+\frac{\mathrm{N1+N3}}{\mathrm{N2}}M_\gamma} \tag{2-13}$$

We found that for desalination polyamides, the HABD calculation formula has the same form.

3. Separation performance of desalination membranes

3.1. Performance of desalination membranes

For both reverse osmosis and nanofiltration polyamide membranes, we use salt rejection, water permeance, solute permeability coefficient, and water permeability to describe the filtration performance.

The salt rejection (R) was determined by:

$$R = \left(1 - \frac{C_p}{C_f}\right) \times 100\% \tag{3-1}$$

Where C_p and C_f are the concentrations of permeated and feed solutions, respectively.

The water permeance (F, L m^{-2} h^{-1} bar^{-1}) of the membranes was calculated by:

$$F = \frac{V}{ATP} \tag{3-2}$$

where V is the volume of permeated solution (L), A is the effective filtration area (m^2), T is the permeation time (h), P is the applied pressure (bar) during the measurement.

The salt permeability coefficient (B, L m^{-2} h^{-1}) of the membranes was calculated by:

$$B = FP\left(\frac{1}{R} - 1\right) \tag{3-3}$$

The water permeability (p, L nm m^{-2} h^{-1} bar^{-1}) of the membranes was calculated by:

$$p = FH \tag{3-4}$$

where H is the thickness of selective polyamide nanofilm (nm).

3.2. Rejection, permeability of NaCl and water permeance in RO and DNC

DNC data in Supplementary Table 3 is directly obtained from reference or calculated by Formula 1 in main text based on the measured element ratio obtained from reference. The salt permeability coefficient in RO can be calculated by Supplementary Formula 3-3 from rejection, water permeance and applied pressure during the measurement.

Supplementary Table 3. Summary of rejection, permeability of NaCl and water permeance in RO and DNC of MPD-TMC polyamide membranes in Fig. 3A and B in main text.

C (%)	O (%)	N (%)	DNC (%)	R (NaCl) (%)	Test conditions	F (L m^{-2} h^{-1} bar^{-1})	B (NaCl) (L m^{-2} h^{-1})	Ref.
76.33	12.8	10.87	**75.5**	95.0	2000 ppm @ 10 bar	5.60	2.95	18
75.44	15.92	11.64	**53.4**	93.0		5.40	4.06	
69.91	17.68	12.41	**47.5**	91.5		4.80	4.46	
75.92	13.23	10.85	**70.3** [a]	98.0	2000 ppm @ 15.5 bar	4.00	1.27	19
75.57	14.08	10.35	**54.2** [a]	97.2		6.20	2.77	
75.36	13.64	11.00	**67.9** [a]	98.0		5.70	1.80	
75.05	14.33	10.62	**55.3** [a]	97.8		3.80	1.32	
75.31	14.49	10.20	**47.9** [a]	97.5		4.90	1.95	
75.08	14.08	10.83	**60.9** [a]	98.9		4.50	0.78	
73.49	16.16	10.36	**34.4**	92.1	1000 ppm @ 8 bar	1.92	1.32	20
73.55	15.19	11.26	**55.4**	94.5		1.86	0.87	
72.97	16.9	10.31	**27.3**	91.2		1.95	1.51	
75.5	13.62	10.88	**66.4**	99.6	35000 ppm @50 bar	1.10	0.22	21
74.9	13.73	11.37	**71.8**	99.4		1.60	0.48	
75.29	13.35	11.36	**75.8**	99.5		1.70	0.43	
79.27	13.45	7.28	**10.7**	98.7		1.20	0.79	
68.9	16.95	9.02	**8.4**	99.0	2000 ppm @ 15 bar	2.80	0.42	22
74.58	14.78	9.71	**37.9**	99.0		5.47	0.83	
73.5	14.28	10.94	**60.3**	98.0		7.00	2.14	
72.76	15.82	9.39	**23.5**	98.0		5.33	1.63	
72.45	15.5	9.5	**28.0**	98.0		5.80	1.78	
72.47	15.13	9.08	**25.0**	98.0		3.47	1.06	
73.53	13.9	9.8	**48.1**	99.0		3.33	0.51	
72	17.2	10.8	**31.4** [a]	99.4	2000 ppm @ 15.5 bar	2.10	0.20	23
73.2	16.1	10.7	**39.6** [a]	99.4		3.00	0.28	

73.1	15.9	11	**45.4** [a]	98.3		4.50	1.21	
73.5	15.4	11.1	**51.3** [a]	95.2		6.30	4.92	
76.9	11.87	11.23	**91.7** [a]	98.8	2000 ppm @ 15.5 bar	0.68	0.13	24
76.74	12.47	10.71	**77.2** [a]	98.7		1.69	0.35	
76.56	12.27	11.17	**85.9** [a]	98.2		1.00	0.28	
72.3	14.6	13.1	**83.8**	97.5	1000 ppm @ 10 bar	1.36	0.35	25
72.1	14.6	13.3	**86.0**	98.3		2.12	0.37	
71.7	15.5	12.8	**71.4**	95.3		2.58	1.27	
72.6	15.3	12.1	**65.0**	94.7		2.76	1.54	
73	15.3	11.7	**60.0**	92.8		2.41	1.87	
77	11.7	11.2	**93.4**	99.1	2000 ppm @ 15 bar	1.73	0.24	26
76.9	11.8	11.4	**94.8**	99.2		2.78	0.34	
68.23	15.03	9.17	**27.4** [a]	98.3	2000 ppm @ 15 bar	2.90	0.75	27
63.71	19.24	8.21	**-20.5** [a]	62.8		18.00	159.94	
74.6	13.1	12.3	**90.6**	98.0	2000 ppm @ 15 bar	1.73	0.53	28
74.4	13.6	12	**81.3**	98.3		1.87	0.48	
74.3	13.8	11.9	**77.8**	98.5		2.07	0.47	
73.7	14.2	12.1	**76.0**	99.0		2.13	0.32	
73.2	14.7	12.1	**70.9**	93.2		2.80	3.06	
72.21	17.51	10.28	**22.0**	94.7	1000 ppm @ 2 bar	1.99	0.22	29
72.7	16.65	10.65	**34.1**	94.0		2.26	0.29	
72.52	16.52	10.96	**39.3**	94.3		2.67	0.32	
77.31	13.36	9.33	**46.7**	95.9		3.44	0.29	
76.76	13.52	9.72	**50.9**	96.7		3.21	0.22	
75.73	13.83	10.44	**58.1**	91.1		2.32	0.45	
77.41	12.12	10.47	**78.1**	98.1	2000 ppm @ 16 bar	3.07	0.95	30
76.28	13.3	10.41	**N. A.**	98.4		3.64	0.95	
75.67	14.23	10.1	**N. A.**	97.3		2.94	1.30	
76.41	14.23	9.36	**38.1**	N. A.	2000 ppm	N.A.	N.A.	31

73.66	14.63	11.71	**66.7**	N.A.	@ 6.8 bar	N.A.	N.A.	
73.59	14.39	12.02	**73.1**	N.A.		N.A.	N.A.	
73.5	14.26	12.24	**77.1**	N.A.		N.A.	N.A.	
75.27	14.7	10.03	**43.3**	N.A.		N.A.	N.A.	
74.84	16.04	9.12	**17.5**	N.A.		N.A.	N.A.	
73.06	15.07	11.24	**56.3**	87.9		2.01	1.89	
70	12	11	**87.0** [a]	97.5	2000 ppm @ 10 bar	2.87	0.74	32
70.13	17.47	11.28	**35.4**	92.6	2000 ppm @ 20 bar	1.40	2.24	33
74.54	15.39	10.07	**37.3**	92.0		1.40	2.43	
73.32	15.33	11.35	**55.2**	94.0		1.60	2.04	
71.44	16.19	12.38	**60.0**	95.8		1.77	1.55	
73.78	14.3	11.92	**72.8**	90.0		2.00	4.44	
73.1	13.1	13.9	**108.9**	99.5	2000 ppm @ 15.5 bar	2.60	0.20	34
76.46	12.75	10.8	**75.2**	98.0	2000 ppm @ 15 bar	1.07	0.33	35
77.14	12.44	10.42	**73.5**	97.0		1.33	0.62	
79.49	13.69	6.82	**-0.5** [a]	98.0		1.60	0.49	
77.99	15.26	6.75	**-16.0** [a]	96.0		1.47	0.92	
77.73	14.86	7.41	**-0.4** [a]	97.0		1.80	0.84	
77.2	12.3	10.4	**74.9** [a]	99.0	35000 ppm @ 55 bar	1.62	0.90	36
77.3	11.8	10.5	**82.5** [a]	99.0		1.17	0.65	
93.36	4.04	2.6	**34.9**	N. A.	2000 ppm @ 15.5 bar	N. A.	N. A.	37
71.09	15.84	13.06	**71.1**	95.0		0.97	0.79	
73.25	13.58	13.17	**95.4**	98.0		3.23	1.02	
75.01	14.71	10.28	**46.8** [a]	97.0	2000 ppm @ 20 bar	1.13	0.70	38
75.21	17.12	10.67	**30.4** [a]	97.9		2.32	1.00	
75.57	13.81	10.62	**60.8** [a]	97.3		3.15	1.75	
75.58	13.74	10.68	**62.4** [a]	97.1		3.31	1.98	

76.35	12.75	10.7	**73.8**	98.9	2000 ppm	1.26	0.21	39
76.28	12.52	11.07	**81.6**	99.2	@ 15 bar	1.68	0.20	
72.1	16.8	11.1	**38.7** [a]	99.6		1.60	0.10	
72.3	17.6	10.1	**18.8** [a]	99.4	2000 ppm	2.50	0.23	
73	16.7	10.3	**28.9** [a]	99.4	@ 15.5 bar	1.90	0.18	40
75.5	14.2	10.3	**52.2** [a]	95.7		10.00	6.96	
73	16.4	10.6	**35.6** [a]	99.6		3.80	0.24	
75.5	10.3	14.2	**147.8** [a]	99.0		0.86	0.13	
75.3	9.7	15	**164.4** [a]	99.0	2000 ppm	0.86	0.13	41
75.5	12.5	12.1	**95.1** [a]	96.7	@ 15.5 bar	0.56	0.30	
75.8	12.9	11.3	**80.2** [a]	95.7		0.81	0.57	
N. A.	N. A.	N. A.	**46.9** [b]	93.6		4.76	4.95	
N. A.	N. A.	N. A.	**38.1** [b]	96.9	2000 ppm	3.16	1.54	42
N. A.	N. A.	N. A.	**37.2** [b]	94.1	@ 15.2 bar	4.99	4.75	
N. A.	N. A.	N. A.	**28.1** [b]	96.2		3.64	2.18	
67.31	17.37	15.32	**81.2** [a]	96.2	1000 ppm	4.03	0.96	43
65.94	18.69	15.36	**70.7** [a]	93.6	@ 6 bar	6.54	2.68	
73.96	17.22	8.82	**3.2** [a]	N. A.		N. A.	N. A.	
75.52	15.6	8.88	**17.6** [a]	N. A.		N. A.	N. A.	
74.85	15.4	9.75	**32.6** [a]	98.8	2000 ppm	3.00	0.55	44
73.57	15.89	10.54	**39.3** [a]	98.8	@ 15 bar	4.00	0.73	
73.35	15.85	10.8	**43.2** [a]	98.8		3.47	0.63	
73.69	15.01	11.3	**57.7** [a]	40.0		0.76	17.10	
70.34	16.45	13.18	**66.9** [a]	99.4		2.84	0.27	
75.47	13.2	11.33	**77.1** [a]	99.3	2000 ppm	3.68	0.40	
74.26	13.45	12.29	**86.5** [a]	99.4	@ 15 bar	4.26	0.40	45
73.93	14.01	12.06	**77.6** [a]	99.4		3.55	0.33	
72.24	15.91	11.85	**56.1** [a]	99.3		3.23	0.35	
76.12	14.98	8.91	**23.8**	94.0	2000 ppm	2.38	2.35	46

70.5	17.26	12.25	**49.1**	96.0	@ 15.5 bar	1.84	1.19	
71.8	16.7	11.1	**39.6**	96.0		2.92	1.89	
75.12	14.84	10.05	**42.3**	N. A.		N. A.	N. A.	
73.7	12.9	13.4	**105.7** [a]	97.5	2000 ppm @ 15.5 bar	2.25	0.89	47
73.8	12.5	13.7	**113.7** [a]	97.5		2.30	0.91	
73.6	12.9	13.5	**106.8** [a]	98.0		2.60	0.82	
74.3	12.8	12.9	**101.2** [a]	97.5		2.60	1.03	
74.1	12.4	13.5	**112.7** [a]	98.0		3.00	0.95	
73.6	12.8	13.6	**109.1** [a]	98.0		2.70	0.85	
75.5	12.5	12.1	**95.1** [a]	99.4	2000 ppm @ 15.3 bar	0.56	0.05	48
73.8	13.3	12.9	**95.4** [a]	96.8		0.84	0.42	
73.54	15.48	10.98	**49.0** [a]	99.0	2000 ppm @ 15.5 bar	2.21	0.35	49
73.48	15.25	11.27	**55.0** [a]	99.0		2.32	0.36	
73.51	15.16	11.33	**56.6** [a]	99.0		2.58	0.40	
73.42	14.89	11.69	**63.9** [a]	99.0		2.90	0.45	
73.4	14.58	12.02	**71.1** [a]	99.0		3.13	0.49	
73.46	14.59	11.59	**65.6** [a]	99.0		2.71	0.42	
74.47	12.92	12.61	**96.4**	94.3	2000 ppm @ 15.2 bar	6.05	5.56	50
72.13	14.93	12.94	**78.6**	97.0		3.49	1.64	
69.61	17.4	12.98	**56.4**	98.5		0.66	0.15	
71.3	17.09	11.61	**42.7**	99.0		1.61	0.25	
73.0	14.7	11.6	**64.6** [a]	92.0	1500 ppm @ 15.1 bar	0.70	0.92	51
74.0	15.6	10.4	**40.0** [a]	98.0	2000 ppm @ 15.5 bar	1.94	0.61	52
72.9	15.5	11.6	**56.8** [a]	98.0		2.71	0.86	
73.1	15.2	11.7	**61.0** [a]	98.0		3.10	0.98	
73.2	15.0	11.8	**64.2** [a]	98.0		3.23	1.02	
73.4	14.8	11.8	**66.2** [a]	97.0		3.23	1.55	

78.47	11.46	10.07	**80.6**	98.2	2000 ppm @ 10 bar	6.80	1.25	[9]
74.27	15.5	10.23	**38.6**	98.3	2000 ppm @ 15 bar	0.80	0.21	[53]
72.46	16.05	11.49	**50.3**	97.4		1.30	0.52	
72.06	16.53	11.41	**45.0**	96.7		1.09	0.56	
72.6	15.57	11.83	**59.1**	97.9		1.64	0.53	
70.33	19.96	9.7	**-3.8**	88.7	2000 ppm @ 8 bar	2.97	3.02	[54]
68.28	22.86	8.85	**-32.5**	93.3		2.57	1.48	
71.06	19.94	9.01	**-13.3**	94.5		2.42	1.13	
68.86	21.89	9.25	**-21.8**	96.2		2.12	0.66	
65.91	27.14	6.96	**-77.5**	97.4		1.89	0.40	
67.53	24.43	8.03	**-51.6**	97.8		1.77	0.31	
65.61	28.27	6.12	**-93.2**	97.0		1.82	0.45	
69.18	21.55	9.28	**-19.4**	97.1		1.73	0.42	
73.64	14.71	11.65	**65.2**	95.0	2000 ppm @ 10 bar	2.30	1.21	[55]
74.33	13.71	11.96	**79.5**	90.0		2.25	2.50	
74.81	13.25	11.93	**84.3**	94.5		2.60	1.51	
74.54	12.92	12.54	**95.5**	98.7		3.05	0.40	
69.63	19.19	10.91	**17.5**	97.20	600 ppm @ 4 bar	6.20	0.71	[56]
69.09	19.77	10.89	**13.1**	97.30		8.30	0.92	
68.31	20.20	10.92	**10.5**	92.30		11.40	3.80	
75.40	13.60	11.00	**68.3** [a]	94.26	2000 ppm @ 10.3 bar	3.85	2.41	[57]
75.50	13.20	11.20	**75.4** [a] [60,61]	99.20 [58,59]	20 mM @ 15.5 bar	1.34	N. A.	SWC4
74.30	12.60	13.20	**107.0** [a]	96.50	10 mM @ 13.8 bar	6.04	N. A.	XLE [60]
74.80	13.10	12.10	**88.1** [a]	95.80		4.29	N. A.	LE [60]
74.30	12.80	12.90	**101.2** [a]	94.90		7.52	N. A.	ESPA3

								[60]
72.90	15.80	11.30	**50.2** [a]	91.50		9.04	N. A.	NE90 [60]
73.90	14.00	12.10	**78.2** [a]	94.40		11.20	N. A.	NF90 [60]

a: This work did not provide the DNC.

b: This work directly provided the DNC without proportion of C, O, and N.

3.3. Rejection, permeability of NaCl and water permeance in RO and HABD

We directly obtained the XPS analysis results from recent researches, including the proportion of C, N, O atoms and the peak splitting results of N1, N2 and N3. We adopted their peak fitting results for N, and excluded the peak fitting results for C or O from the cited works due to their lack of consideration for the cross-peak self-consistency relationship between atoms, such as the equality of N2 and O2. In our calculation of HABD, we directly equated O2 in these works to N2. The remaining portion obtained by subtracting O2 from the total O is assumed to represent O1 of polyamide. The HABD of MPD-TMC polyamide is calculated by Formula 2 in main text based on the N1, N2, N3 and O1 form corresponding references.

Supplementary Table 4. Summary of rejection, permeability of NaCl and water permeance in RO and HABD of MPD-TMC polyamide membranes in Fig. 3C and D in main text.

**C (%)**	**O (%)**	**N (%)**	**N1+ N3 (%)**	**N2 (%)**	**O1 (%)**	**HABD (mmol g^{-1})**	**R (NaCl) (%)**	**F (L m^{-2} h^{-1} bar^{-1})**	**B (NaCl) (L m^{-2} h^{-1})**	**Ref.**
76.9	11.87	11.23	0	11.23	0.64	**9.25**	98.8	0.68	0.13	
76.74	12.47	10.71	0.32	10.38	2.08	**8.71**	98.7	1.69	0.35	[24]
76.56	12.27	11.17	0	11.17	1.10	**9.13**	98.2	1.00	0.28	
77	11.7	11.2	0.38	10.82	0.88	**9.02**	99.1	1.73	0.24	
76.9	11.8	11.4	0.36	11.04	0.76	**9.06**	99.2	2.78	0.34	[26]
76.28	13.3	10.41	N. A.	N. A.	N. A.	**N. A.**	98.4	3.64	0.95	
75.67	14.23	10.1	N. A.	N. A.	N. A.	**N. A.**	97.3	2.94	1.30	
73.1	13.1	13.9	1.28	12.62	0.48	**8.86**	99.5	2.60	0.20	[34]

76.35	12.75	10.7	0.92	9.78	2.97	**8.20**	98.9	1.26	0.21	39
76.28	12.52	11.07	1.10	9.97	2.55	**8.26**	99.2	1.68	0.20	
78.47	11.46	10.07	0.8	9.27	2.19	**8.40**	98.2	6.80	1.25	9
70.33	19.96	9.7	1.17	8.53	11.43	**6.23**	88.7	2.97	3.02	54
68.28	22.86	8.85	1.45	7.40	15.46	**5.27**	93.3	2.57	1.48	
71.06	19.94	9.01	0.71	8.30	11.64	**6.26**	94.5	2.42	1.13	
68.86	21.89	9.25	0.9	8.35	13.54	**5.93**	96.2	2.12	0.66	
65.91	27.14	6.96	0.63	6.33	20.81	**4.41**	97.4	1.89	0.40	
67.53	24.43	8.03	0.77	7.26	17.17	**5.14**	97.8	1.77	0.31	
65.61	28.27	6.12	0.5	5.62	22.65	**3.97**	97.0	1.82	0.45	
69.18	21.55	9.28	0.97	8.31	13.24	**5.94**	97.1	1.73	0.42	
73.64	14.71	11.65	1.86	9.79	4.92	**7.46**	95.0	2.30	1.21	55
74.33	13.71	11.96	2.16	9.80	3.91	**7.58**	90.0	2.25	2.50	
74.81	13.25	11.93	1.99	9.94	3.31	**7.78**	94.5	2.60	1.51	
74.54	12.92	12.54	2.51	10.03	2.89	**7.71**	98.7	3.05	0.40	
69.63	19.19	10.91	0.66	8.73	10.46	**6.57**	97.20	6.20	0.71	56
69.09	19.77	10.89	0.58	8.71	11.06	**6.49**	97.30	8.30	0.92	
68.31	20.20	10.92	1.42	8.74	11.46	**6.22**	92.30	11.40	3.80	

3.4. Correlation between NaCl permeability in RO with HABD or DNC

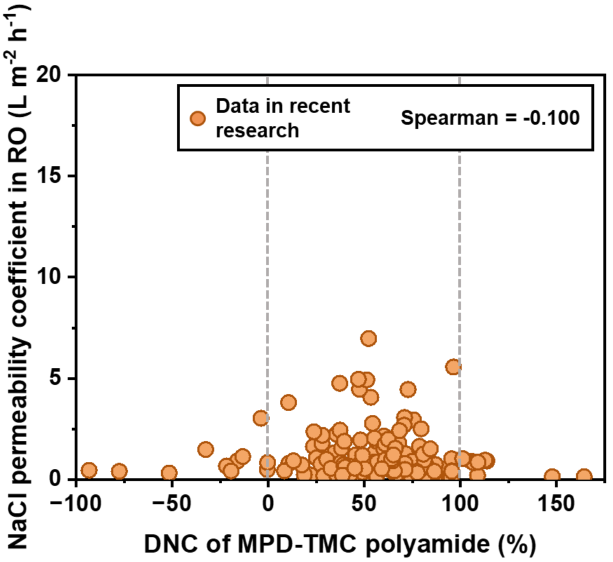

Supplementary Fig. 9. The correlation analysis between DNC and NaCl permeability coefficient in RO.

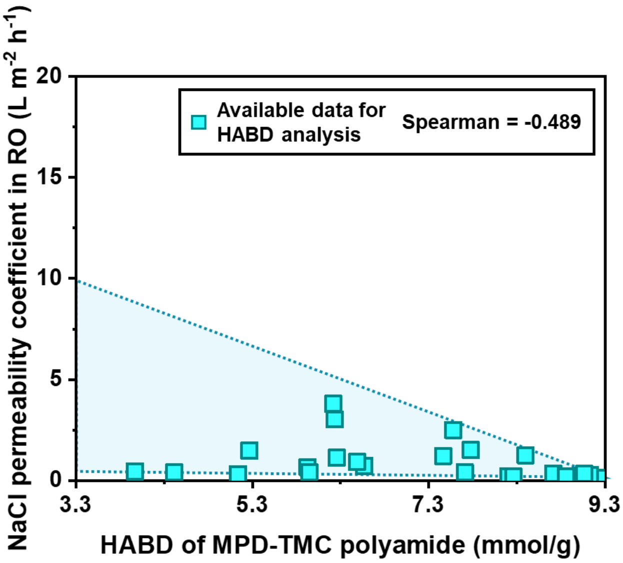

Supplementary Fig. 10. The correlation analysis between HABD and NaCl permeability coefficient in RO.

3.5. Rejection, permeability of Na_2SO_4 and water permeance in NF and DNC

DNC data in Supplementary Table 5 is directly obtained from reference or calculated by Formula 1 in main text based on the measured element ratio obtained from reference. The salt

permeability coefficient in NF can be calculated by Supplementary Formula 3-3 from rejection, water permeance and applied pressure during the measurement.

Supplementary Table 5. Summary of rejection, permeability of Na_2SO_4 and water permeance in NF and DNC of PIP-TMC polyamide membranes in Fig. 4A and B in main text.

**C (%)**	**O (%)**	**N (%)**	**DNC (%)**	**R (Na_2SO_4) (%)**	**Test conditions**	**F (L m^{-2} h^{-1} bar^{-1})**	**B (Na_2SO_4) (L m^{-2} h^{-1})**	**Ref.**
72.1	16.4	11.5	**47.3**	99.0	1000 ppm @ 6 bar	7.4	0.45	62
72.4	16	11.6	**52.2**	99.0		13.9	0.84	
72.9	15.5	11.6	**56.8**	99.0		16.8	1.02	
73.9	14.6	11.5	**64.4**	99.0		25.2	1.53	
73.2	15.3	11.5	**57.5**	99.0		21.4	1.30	
70.98	15.63	13.16	**74.3**	96.5	1000 ppm @ 6 bar	8.1	1.76	63
70.46	16.15	13.16	**69.4**	96.6		26.5	5.60	
73.35	15.25	11.4	**56.7**	99.0	1000 ppm @ 6 bar	12.0	0.73	64
71.5	15.49	12.5	**68.0**	98.0		17.0	2.08	
69.3	16.21	13.2	**69.3**	95.8		20.0	5.26	
74.45	13.84	11.19	**68.2**	91.0	1000 ppm @ 8 bar	0.6	0.49	65
74.28	14.32	11.23	**63.7**	94.0		0.9	0.45	
73.78	14.19	11.88	**73.4**	90.0		1.1	1.00	
71.7	16.15	11.96	**55.3**	85.0		1.6	2.29	
75.03	12.46	12.51	**100.6**	78.0		2.3	5.08	
71.4	15.02	13.58	**84.9**	92.2	500 ppm @ 5 bar	4.9	2.07	66
68.73	16.42	14.46	**81.0**	N. A.		N. A.	N. A.	
69.56	15.5	13.4	**78.2**	91.0		9.6	4.75	
73.87	15.35	10.78	**47.5**	99.1	2000 ppm @ 5 bar	9.1	0.41	67
72.19	17.05	10.76	**32.1**	98.4		21.0	1.70	
70.36	16.13	12.98	**67.5**	98.0	20 mM @ 4	26.0	2.12	68

68.1	16.72	14.79	**81.6**	98.5	bar	30.0	1.83	
70.39	16.64	12.66	**59.2**	93.0		28.0	8.43	
70.66	15.73	13.11	**72.7**	92.0		8.0	2.78	
70.38	16.25	12.95	**66.1**	95.0		10.0	2.11	
69.22	16.6	13.91	**73.5**	98.1		24.0	1.86	
70.97	18.15	10.88	**24.9**	98.0	1000 ppm @ 5 bar	5.3	0.54	69
76.42	15.03	8.55	**17.6**	79.7		9.3	11.84	
69.21	19.61	11.18	**17.9**	90.0		7.0	3.89	
73.81	16.38	9.81	**24.7**	93.0		4.8	1.81	
79.31	13.37	7.16	**9.3**	84.0		2.5	2.38	
68.51	18.94	10.29	**11.2**	54.0	1000 ppm @ 5 bar	42.5	181.02	70
69.11	19.08	11.08	**20.4**	91.0		25.0	12.36	
69.46	18.13	12.17	**41.0**	94.0		23.0	7.34	
72.26	14.35	11.73	**69.9**	98.0		20.0	2.04	
69.73	15.09	14.32	**92.1**	92.0		19.5	8.48	
72.86	16.09	11.05	**44.3**	98.1	1000 ppm @ 2 bar	46.6	1.81	71
71.19	17.62	11.2	**33.2**	92.0		55.0	9.57	
69.65	18.19	12.17	**40.5**	97.2	2000 ppm @ 10 bar	7.5	2.15	72
70.21	16.73	13.06	**63.0**	99.2		13.4	1.08	
69.06	19.71	11.23	**17.8**	97.8		9.2	2.07	
69.05	18.99	11.95	**31.7**	98.4		15.4	2.50	
70.01	16.93	13.06	**61.3**	98.8	1000 ppm @ 10 bar	4.8	0.58	73
73.14	14.87	11.99	**67.8**	97.0		7.0	2.16	
66.53	18.51	14.96	**68.2**	96.0		10.0	4.17	
70.92	16.08	13	**68.2**	98.7		14.5	1.91	
67.35	17.75	14.9	**73.8**	94.7		20.6	11.53	
77.42	14.46	8.12	**15.8** [a]	95.0	1000 ppm @ 8 bar	3.0	1.26	74
80.38	17.46	2.15	**-134.2** [a]	34.0		21.0	326.12	
77.63	16.24	6.13	**-35.6** [a]	95.0		9.3	3.92	

78.22	13.87	7.91	**17.9** [a]	97.0		7.0	1.73	
76.64	13.67	9.69	**48.9** [a]	98.0		5.0	0.82	
71.8	17.44	10.76	**28.9**	95.0	1000 ppm @ 4 bar	17.0	3.58	75
73.75	15.86	10.47	**38.6**	97.0		23.0	2.85	
74.48	14.85	10.26	**45.2**	96.0		25.5	4.25	
73.41	15.49	10.54	**43.0**	93.5		26.0	7.23	
71.48	16.04	11.52	**50.8**	97.5		30.5	3.13	
53.88	28.43	17.69	**30.1** [a]	97.6	N. A.	38.2	N. A.	76
73.82	14.7	11.48	**63.1**	99.0	1000 ppm @ 4 bar	6.8	0.27	77
73.45	14.91	11.64	**63.1**	97.0		14.2	1.75	
73.12	15.14	11.74	**62.1**	96.0		17.0	2.83	
72.9	15.42	11.68	**58.6**	95.5		21.2	4.00	
71.24	16.78	11.88	**48.7**	86.0		28.7	18.67	
70.3	19.89	9.82	**-1.7** [a]	92.0	1000 ppm @ 4.8 bar	26.0	10.87	78
70.29	19.12	10.59	**13.9** [a]	95.5		41.7	9.42	
73.61	15.18	11.21	**54.9**	94.0	2000 ppm @ 10 bar	6.8	4.34	79
71.74	16.37	11.89	**52.4**	98.5		13.0	1.98	
70.5	16.67	12.83	**60.9**	98.5		17.0	2.59	
72.82	14.9	12.28	**71.1**	98.5		16.5	2.51	
74.69	13.69	11.62	**75.5**	98.5		15.0	2.28	
87.18	6.93	5.69	**70.5**	91.3	2000 ppm @ 5 bar	6.5	3.10	80
77.49	12.29	9.86	**67.1**	96.4		8.5	1.59	
73.95	13.8	12.25	**82.1**	99.6	N. A.	22.0	N. A.	81
73.82	13.8	12.38	**83.7**	99.6		48.0	N. A.	
73.73	13.9	12.37	**82.5**	99.6		35.0	N. A.	
74.05	13.7	12.25	**83.2**	99.6		32.0	N. A.	
69.8	15.2	14.5	**92.9**	87.6	1000 ppm @ 2 bar	50.2	14.26	82

66.82	17.67	10.58	**24.7** [a]	99.5	2000 ppm @ 6 bar	27.0	0.81	27
56.37	20.52	8.91	**-18.3** [a]	87.5		36.5	31.29	
76.77	13.79	9.43	**43.7**	90.8	1000 ppm @ 6 bar	26.6	16.17	83
74.5	15.83	9.67	**27.5**	96.0		25.0	6.25	
74.6	16.2	9.2	**17.3**	95.3		28.2	8.34	
75.08	16.08	8.84	**12.8**	95.6		32.3	8.93	
70.26	20.24	9.5	**-8.3** [a]	N. A.	N. A.	N. A.	N. A.	84
68.74	21.76	9.5	**-17.7** [a]	N. A.		N. A.	N. A.	
70.04	21.07	8.89	**-22.0** [a]	94.0		50.0	19.15	
69.06	21.92	9.01	**-25.2** [a]	91.0		55.0	32.64	
70.51	17.34	12.15	**47.2** [a]	N. A.	1000 ppm @ 6 bar	N. A.	N. A.	85
67.72	21.68	10.6	**-3.0** [a]	97.5		14.5	2.23	
67.62	21.47	10.91	**2.2** [a]	97.8		15.8	2.14	
68.38	19.82	11.8	**23.9** [a]	98.2		20.0	2.20	
69.01	19.42	11.57	**24.0** [a]	97.4		15.0	2.40	
68.81	19.07	12.12	**33.2** [a]	98.2		18.3	2.02	
69.65	17.15	13.19	**46.9** [a]	98.4		23.3	2.28	
71.25	15.69	13.06	**38.1**	98.0	2000 ppm @ 6 bar	16.5	2.02	86
71.24	15.52	13.24	**37.2**	98.0		16.0	1.96	
71.33	15.38	13.29	**28.1**	97.0		15.0	2.78	
71.28	14.78	13.94	**91.2**	90.0		11.0	7.33	
71.01	14.7	14.29	**95.8**	31.0		24.0	320.52	
73	16	11	**44.4**	99.0	2000 ppm @ 10 bar	8.0	0.81	87
78.6	12	9.4	**63.6**	99.0		24.0	2.42	
71.65	16.38	11.97	**53.3**	98.4	2000 ppm @ 10 bar	8.4	1.37	88
71.93	15.95	12.12	**59.1**	99.2		9.3	0.75	
71.9	15.72	12.38	**64.3**	99.5		11.7	0.59	
71.89	15.47	12.64	**69.8**	99.5		13.5	0.68	
72.07	14.96	12.97	**78.6**	99.2		16.8	1.35	

72.8	15.11	13.01	**77.6**	99.0		16.9	1.71	
78.2	11.8	10	**75.2** [a]	79.0	1000 ppm @ 7.6 bar	3.3	6.65	89
74.8	13.8	11.4	**71.4** [a]	80.0		5.3	10.00	
75.5	13.9	10.6	**59.6** [a]	95.0		6.1	2.42	
76.2	12.7	11.1	**79.8** [a]	96.0		7.5	2.38	
73.81	14.39	11.8	**70.3**	97.0	1000 ppm @ 4 bar	13.4	1.66	90
76.54	13.07	10.49	**67.1**	98.0		24.0	1.96	
70.22	17.56	11.71	**40.0**	97.0		27.6	3.41	
73.32	16.15	10.33	**34.1**	93.9		48.9	12.71	
78.28	11.75	9.97	**75.4** [a]	96.5	1000 ppm @ 6 bar	40.0	8.70	91
72.45	17.45	10.1	**20.0** [a]	96.0	2000 ppm @ 6 bar	6.3	1.58	92
74.8	14.51	10.69	**54.5** [a]	93.0		1.7	0.75	
70.93	16.92	12.15	**50.8**	94.0	1000 ppm @ 6 bar	3.8	1.47	93
70.92	15.68	13.4	**76.5**	97.0		9.3	1.73	
73.95	15.34	10.33	**41.4**	90.0	1000 ppm @ 4 bar	10.4	4.62	94
72.18	15.58	11.55	**55.4**	94.0		14.5	3.70	
70.68	15.98	12.32	**61.2**	97.0		21.5	2.66	
70.02	16.12	12.78	**65.3**	96.5		23.9	3.47	
71.35	15.86	12.79	**67.9**	99.7	1000 ppm @ 6 bar	13.8	0.29	95
71.85	15.7	12.45	**65.4**	99.6		14.9	0.40	
71.24	16.48	12.29	**56.3**	99.5		17.5	0.57	
70.54	17.79	11.66	**37.6**	99.4		20.9	0.76	
71.87	17.05	11.08	**36.3**	97.8		24.5	3.27	
71.55	14.54	13.91	**93.4**	99.6	1000 ppm @ 6 bar	20.0	0.48	96
72.63	14.82	12.82	**78.3**	98.8		17.5	1.28	
71.71	14.71	13.59	**88.1**	98.0		20.0	2.45	
75.26	21.16	3.59	**-113.0** [a]	98.0	1000 ppm @ 6 bar	7.5	0.92	97
75.08	19.17	5.75	**-61.6** [a]	97.6		17.5	2.58	

75.94	17.69	6.37	**-41.1** [a]	94.2		12.7	4.68	
76.46	17.04	6.5	**-34.3** [a]	95.8		10.5	2.76	
76.63	17.65	6.72	**-34.6** [a]	94.8		8.7	2.85	
73.12	16.65	10.23	**28.3** [a]	N. A.	1000 ppm @ 6 bar	N. A.	N. A.	[98]
70.54	20.23	9.23	**-12.0** [a]	95.2		34.0	10.29	
71.72	18.14	10.14	**15.1** [a]	97.0		18.0	3.34	
74.74	15.4	9.85	**34.1** [a]	98.0		10.0	1.22	
67.35	18.45	9.84	**8.7** [a]	98.6	2000 ppm @ 2 bar	45.5	1.29	[99]
71.20	16.40	12.50	**59.5** [60]	95.20 [100]	2000 ppm @ 4 bar	9.43	1.90	NF 270

a: This work did not provide the DNC.

3.6. Rejection, permeability of Na_2SO_4 and water permeance in NF and HABD

We directly obtained the XPS analysis results from recent researches, including the proportion of C, N, O atoms and the peak splitting results of N1, N2 and N3. We adopted their peak fitting results for N, and excluded the peak fitting results for C or O from the cited works due to their lack of consideration for the cross-peak self-consistency relationship between atoms, such as the equality of N2 and O2. In our calculation of HABD, we directly equated O2 in these works to N2. The remaining portion obtained by subtracting O2 from the total O is assumed to represent O1 of polyamide. The HABD of PIP-TMC polyamide is calculated by Formula 2 in main text based on the N1, N2, N3 and O1 form corresponding references.

Supplementary Table 6. Summary of rejection, permeability of Na_2SO_4 and water permeance in NF and HABD of PIP-TMC polyamide membranes in Fig. 4C and D in main text.

**C (%)**	**O (%)**	**N (%)**	**N1+ N3 (%)**	**N2 (%)**	**O1 (%)**	**HABD (mmol g^{-1})**	**R (Na_2SO_4) (%)**	**F (L m^{-2} h^{-1} bar^{-1})**	**B (Na_2SO_4) (L m^{-2} h^{-1})**	**Ref.**
70.98	15.63	13.16	0.33	12.83	2.80	**9.63**	96.5	8.1	1.76	[63]

70.46	16.15	13.16	0.36	12.80	3.35	**9.48**	96.6	26.5	5.60	
73.35	15.25	11.4	2.97	8.43	7.06	**7.16**	99.0	12.0	0.73	64
71.5	15.49	12.5	3.54	8.96	7.25	**7.12**	98.0	17.0	2.08	
69.3	16.21	13.2	4.09	9.11	4.73	**7.54**	95.8	20.0	5.26	
69.65	18.19	12.17	0.16	12.90	3.83	**9.43**	97.2	7.5	2.15	72
70.21	16.73	13.06	0.07	11.16	8.55	**8.19**	99.2	13.4	1.08	
69.06	19.71	11.23	0.30	11.65	7.34	**8.46**	97.8	9.2	2.07	
69.05	18.99	11.95	0.16	12.90	4.03	**9.38**	98.4	15.4	2.50	
71.8	17.44	10.76	2.25	8.22	7.64	**7.17**	95.0	17.0	3.58	75
73.75	15.86	10.47	2.06	8.20	6.65	**7.44**	97.0	23.0	2.85	
74.48	14.85	10.26	2.36	8.18	7.31	**7.20**	96.0	25.5	4.25	
73.41	15.49	10.54	2.21	9.31	6.73	**7.65**	93.5	26.0	7.23	
71.48	16.04	11.52	0.93	16.76	11.67	**8.20**	97.5	30.5	3.13	
70.3	19.89	9.82	N. A.	N. A.	N. A.	**7.31**[a]	92.0	26.0	10.87	78
70.29	19.12	10.59	N. A.	N. A.	N. A.	**7.86**[a]	95.5	41.7	9.42	
73.61	15.18	11.21	0.31	11.58	4.79	**9.03**	94.0	6.8	4.34	79
71.74	16.37	11.89	N. A.	N. A.	N. A.	**N. A.**	98.5	13.0	1.98	
70.5	16.67	12.83	0.40	11.88	3.02	**9.48**	98.5	17.0	2.59	
72.82	14.9	12.28	N. A.	N. A.	N. A.	**N. A.**	98.5	16.5	2.51	
74.69	13.69	11.62	N. A.	N. A.	N. A.	**N. A.**	98.5	15.0	2.28	
87.18	6.93	5.69	0.30	5.39	1.54	**9.31**	91.3	6.5	3.10	80
77.49	12.29	9.86	0.38	9.48	2.81	**9.33**	96.4	8.5	1.59	
70.26	20.24	9.5	2.40	7.10	13.14	**5.73**	N. A.	N. A.	N. A.	84
68.74	21.76	9.5	1.95	7.55	14.21	**5.81**	N. A.	N. A.	N. A.	
70.04	21.07	8.89	2.11	6.78	14.29	**5.49**	94.0	50.0	19.15	
69.06	21.92	9.01	2.30	6.71	15.21	**5.29**	91.0	55.0	32.64	
71.65	16.38	11.97	N. A.	N. A.	N. A.	**7.60**[a]	98.4	8.4	1.37	88
71.93	15.95	12.12	N. A.	N. A.	N. A.	**7.81**[a]	99.2	9.3	0.75	
71.9	15.72	12.38	N. A.	N. A.	N. A.	**7.99**[a]	99.5	11.7	0.59	

71.89	15.47	12.64	N. A.	N. A.	N. A.	**8.13** [a]	99.5	13.5	0.68	
72.07	14.96	12.97	N. A.	N. A.	N. A.	**8.40** [a]	99.2	16.8	1.35	
72.8	15.11	13.01	N. A.	N. A.	N. A.	**8.19** [a]	99.0	16.9	1.71	
71.35	15.86	12.79	2.05	10.74	5.12	**8.33**	99.7	13.8	0.29	95
71.85	15.7	12.45	2.33	10.12	5.58	**8.04**	99.6	14.9	0.40	
71.24	16.48	12.29	2.20	10.09	6.39	**7.90**	99.5	17.5	0.57	
70.54	17.79	11.66	2.58	9.08	8.71	**7.10**	99.4	20.9	0.76	
71.87	17.05	11.08	5.86	5.22	11.83	**4.49**	97.8	24.5	3.27	
71.55	14.54	13.91	2.22	11.69	2.85	**8.94**	99.6	20.0	0.48	96
72.63	14.82	12.82	2.28	10.54	4.28	**8.43**	98.8	17.5	1.28	
71.71	14.71	13.59	1.82	11.77	2.94	**9.05**	98.0	20.0	2.45	
73.12	16.65	10.23	3.21	7.02	9.63	**6.14**	N. A.	N. A.	N. A.	98
70.54	20.23	9.23	2.82	6.41	13.82	**5.28**	95.2	34.0	10.29	
71.72	18.14	10.14	3.77	6.37	11.77	**5.39**	97.0	18.0	3.34	
74.74	15.4	9.85	2.92	6.93	8.47	**6.41**	98.0	10.0	1.22	
67.35	18.45	9.84	3.58	6.26	12.19	**5.32**	98.6	45.5	1.29	99

a: This work directly provided the proportion of carboxyl group, amide bond, and amino group.

3.7. Correlation between Na_2SO_4 permeability in NF and DNC or HABD

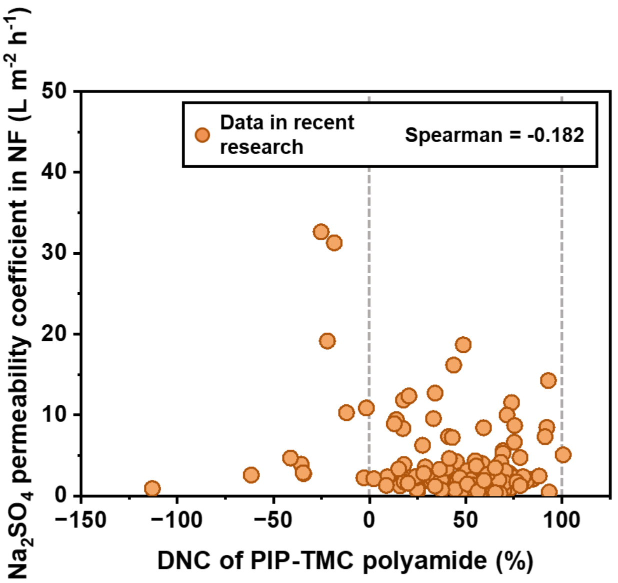

Supplementary Fig. 11. The correlation analysis between DNC and Na_2SO_4 permeability coefficient in NF.

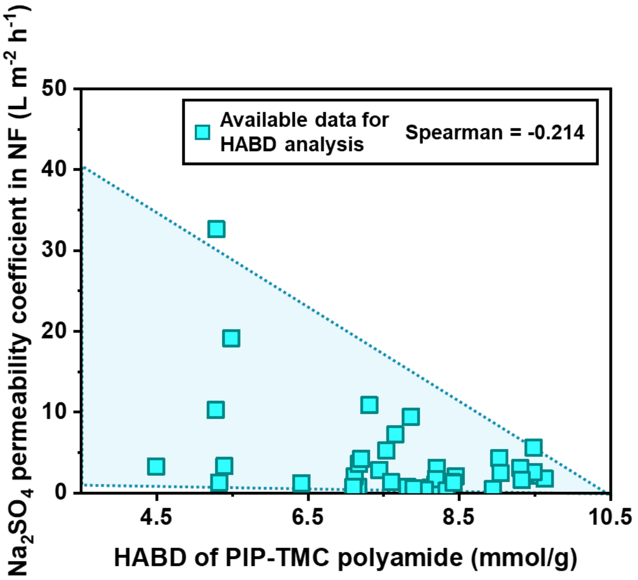

Supplementary Fig. 12. The correlation analysis between HABD and Na_2SO_4 permeability coefficient in NF.

3.8. Water permeability in RO

The water permeability in RO can be calculated by Supplementary Formula 3-4 from thickness of polyamide and water permeance during the measurement.

Supplementary Table 7. Summary of thickness, DNC and HABD of MPD-TMC polyamide nanofilms and RO water permeability of high-performance membranes.

DNC (%)	HABD (mmol g^{-1})	F (L m^{-2} h^{-1} bar^{-1})	Thickness (nm)	Thickness testing method and equipment	P (L nm m^{-2} h^{-1} bar^{-1})	Ref.
75.5	**N. A.**	5.60	148.7	SEM, Inspect F, FEI	832.72	18
53.4	**N. A.**	5.40	163.7		883.98	
47.5	**N. A.**	4.80	172.3		827.04	
70.3	**N. A.**	4.00	68	FESEM, JEOL	272.00	19
54.2	**N. A.**	6.20	55		341.00	
67.9	**N. A.**	5.70	147		837.90	
55.3	**N. A.**	3.80	136		516.80	
47.9	**N. A.**	4.90	197		965.30	
60.9	**N. A.**	4.50	177		796.50	
34.4	**N. A.**	1.92	299	FESEM, JSM-7600F	574.08	20
55.4	**N. A.**	1.86	346		643.56	
27.3	**N. A.**	1.95	292		569.40	
66.4	**N. A.**	1.10	31	FESEM, JEOL JSM7200F	34.10	21
71.8	**N. A.**	1.60	24		38.40	
75.8	**N. A.**	1.70	20		34.00	
10.7	**N. A.**	1.20	24		28.80	
31.4	**N. A.**	2.10	217.2	SEM, FEI Inspect F50	456.12	23
39.6	**N. A.**	3.00	214.1		642.30	
45.4	**N. A.**	4.50	216.7		975.15	
51.3	**N. A.**	6.30	212.1		1336.23	
91.7	**9.25**	0.68	141	FESEM, Carl Zeiss, SUPRA 55VP	95.88	24
77.2	**8.71**	1.69	83		140.27	
85.9	**9.13**	1.00	71		71.00	
83.8	**N. A.**	1.36	139	FESEM, JSF-7500F,	189.04	25

86.0	**N. A.**	2.12	122	JEOL	258.64	
71.4	**N. A.**	2.58	154		397.32	
65.0	**N. A.**	2.76	179		494.04	
60.0	**N. A.**	2.41	194		467.54	
27.4	**N. A.**	2.90	6.7	AFM, Shimadzu SPM-9700	19.43	27
-20.5	**N. A.**	18.00	3.3		59.40	
90.6	**N. A.**	1.73	20	SEM, HitachiS-4300	34.67	28
81.3	**N. A.**	1.87	20		37.33	
77.8	**N. A.**	2.07	70		144.67	
76.0	**N. A.**	2.13	150		320.00	
70.9	**N. A.**	2.80	250		700.00	
78.1	**N. A.**	3.07	186	TEM, Tecnai G2 F20, FEI	571.83	30
N. A.	**N. A.**	3.64	171		621.69	
N. A.	**N. A.**	2.94	147		431.81	
108.9	**8.86**	2.60	7.2	AFM, NX10, Park systems	18.72	34
46.8	**N. A.**	1.13	40	SEM, Nova NanoSEM	45.20	38
30.4	**N. A.**	2.32	13.6		31.55	
60.8	**N. A.**	3.15	12.8		40.32	
62.4	**N. A.**	3.31	11.5		38.07	
73.8	**8.20**	1.26	425	TEM, Hitachi HT-7700	536.20	39
81.6	**8.26**	1.68	150		251.70	
38.7	**N. A.**	1.60	331	SEM, FEI Inspect F50	529.60	40
18.8	**N. A.**	2.50	319		797.50	
28.9	**N. A.**	1.90	328		623.20	
52.2	**N. A.**	10.00	320		3200.00	
35.6	**N. A.**	3.80	330		1254.00	
147.8	**N. A.**	0.86	N. A.	AFM, Veeco Nanoscope V	N. A.	41
164.4	**N. A.**	0.86	17		14.59	

95.1	**N. A.**	0.56	80		44.90	
80.2	**N. A.**	0.81	32		26.01	
49.0	**N. A.**	2.21	458	SEM, Hitachi S-4800	1012.92	49
55.0	**N. A.**	2.32	375		870.97	
56.6	**N. A.**	2.58	250		645.16	
63.9	**N. A.**	2.90	237		688.06	
71.1	**N. A.**	3.13	158		495.10	
65.6	**N. A.**	2.71	152		411.87	
40.0	**N. A.**	1.94	175	SEM, Nova NanoSEM 430	338.71	52
56.8	**N. A.**	2.71	225		609.68	
61.0	**N. A.**	3.10	250		774.19	
64.2	**N. A.**	3.23	280		903.23	
66.2	**N. A.**	3.23	300		967.74	
80.6	**8.40**	6.80	15	AFM, MultiMode, Vecco	102.00	9
-3.8	**6.23**	2.97	8.6	SPM, Veeco MultiMode	25.56	54
-32.5	**5.27**	2.57	8.6		22.06	
-13.3	**6.26**	2.42	10.1		24.39	
-21.8	**5.93**	2.12	12.8		27.18	
-77.5	**4.41**	1.89	10.4		19.69	
-51.6	**5.14**	1.77	12.6		22.29	
-93.2	**3.97**	1.82	11.8		21.50	
-19.4	**5.94**	1.73	12		20.81	
65.2	**7.46**	2.30	50	AFM, Veeco, MultiMode	115.00	55
79.5	**7.58**	2.25	120		270.00	
84.3	**7.78**	2.60	75		195.00	
95.5	**7.71**	3.05	45		137.25	

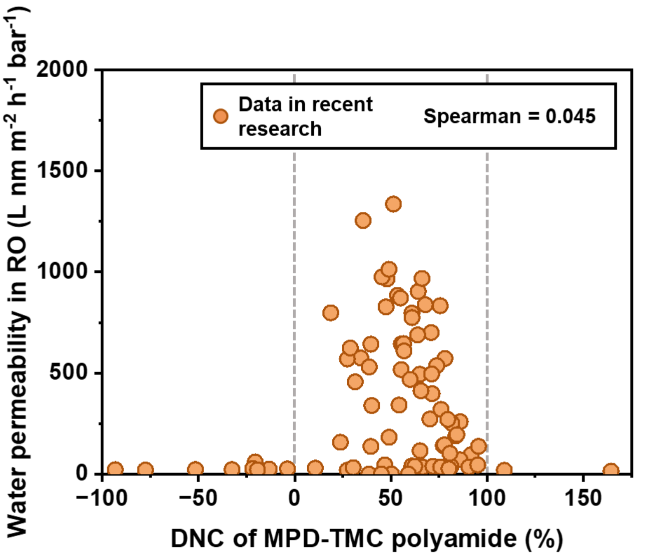

Supplementary Fig. 13. The correlation analysis between DNC and water permeability in RO.

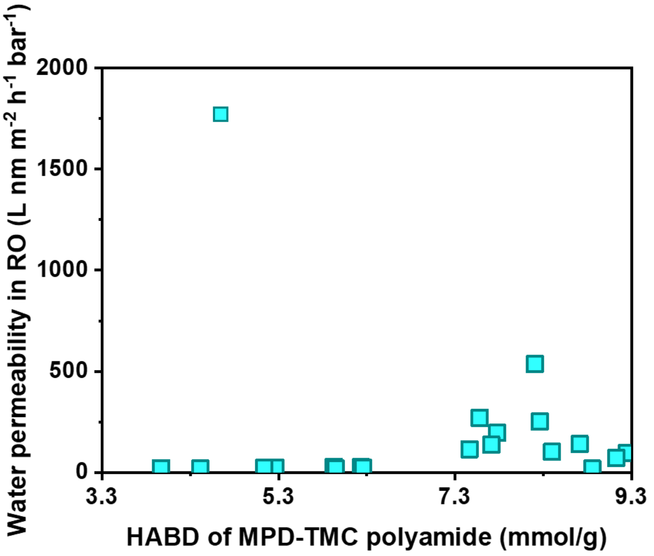

Supplementary Fig. 14. The correlation analysis between HABD and water permeability in RO.

3.9. Water permeability in NF

The water permeability in NF can be calculated by Supplementary Formula 3-4 from thickness of polyamide and water permeance during the measurement.

Supplementary Table 8. Summary of thickness, DNC and HABD of PIP-TMC polyamide nanofilms and NF water permeability of high-performance membranes.

**DNC (%)**	**HABD (mmol g^{-1})**	**F (L m^{-2} h^{-1} bar^{-1})**	**Thickness (nm)**	**Thickness testing method and equipment**	**P (L nm m^{-2} h^{-1} bar^{-1})**	**Ref.**
47.3	**N. A.**	7.4	33.4	FESEM, NOVA NANOSEM 450	247.16	62
52.2	**N. A.**	13.9	28.8		400.32	
56.8	**N. A.**	16.8	23.5		394.80	
64.4	**N. A.**	25.2	18.2		458.64	
57.5	**N. A.**	21.4	20.1		430.14	
56.7	**7.16**	12.0	166.6	SEM, S-4800	1999.20	64
68.0	**7.12**	17.0	44.5		756.50	
69.3	**7.54**	20.0	27		540.00	
68.2	**N. A.**	0.6	220.8	FESEM, Hitachi S-4800	138.00	65
63.7	**N. A.**	0.9	140.2		122.68	
73.4	**N. A.**	1.1	104.9		118.01	
55.3	**N. A.**	1.6	86.6		140.73	
100.6	**N. A.**	2.3	67		150.75	
84.9	**N. A.**	4.9	568	SEM, S4800, Hitachi	2783.20	66
81.0	**N. A.**	N. A.	468		N. A.	
78.2	**N. A.**	9.6	235		2256.00	
47.5	**N. A.**	9.1	75	FE-SEM, SU8010, Hitachi	679.50	67
32.1	**N. A.**	21.0	64		1341.44	
24.9	**N. A.**	5.3	N. A.	TEM, Tecnai F20	N. A.	69
17.6	**N. A.**	9.3	25		232.50	
17.9	**N. A.**	7.0	48		336.00	
24.7	**N. A.**	4.8	75		360.00	
9.3	**N. A.**	2.5	98		245.00	
44.3	**N. A.**	46.6	8.5	AFM, Agilent5500	396.10	71

33.2	**N. A.**	55.0	8.8		484.00	
61.3	**N. A.**	4.8	79.3	FETEM, JEM 2100 F	380.64	73
67.8	**N. A.**	7.0	171.5		1200.50	
68.2	**N. A.**	10.0	180.2		1802.00	
68.2	**N. A.**	14.5	243.5		3530.75	
73.8	**N. A.**	20.6	269.9		5559.94	
15.8	**N. A.**	3.0	164	SEM, Hitachi SU8020,	492.00	74
-134.2	**N. A.**	21.0	87		1827.00	
-35.6	**N. A.**	9.3	94		874.20	
17.9	**N. A.**	7.0	129		903.00	
48.9	**N. A.**	5.0	143		715.00	
28.9	**7.17**	17.0	87	SEM, CSPM5500, Being Nano-Instruments,	1479.00	75
38.6	**7.44**	23.0	100		2300.00	
45.2	**7.20**	25.5	125		3187.50	
43.0	**7.65**	26.0	63		1638.00	
50.8	**8.20**	30.5	42		1281.00	
63.1	**N. A.**	6.8	28.6	FESEM, NOVA NANOSEM 450	194.48	77
63.1	**N. A.**	14.2	26.6		376.39	
62.1	**N. A.**	17.0	22.6		383.64	
58.6	**N. A.**	21.2	21.5		456.34	
48.7	**N. A.**	28.7	16.8		481.74	
54.9	**9.03**	6.8	106	AFM, SPM-9700	720.80	79
52.4	**N. A.**	13.0	82		1066.00	
60.9	**9.48**	17.0	45		765.00	
71.1	**N. A.**	16.5	42		693.00	
75.5	**N. A.**	15.0	38		570.00	
70.5	**9.31**	6.5	45	TEM, HT-7700, Hitachi	292.50	80
67.1	**9.33**	8.5	20		170.00	

92.9	**N. A.**	50.2	80	TEM	4016.00	82
24.7	**N. A.**	27.0	13.4	AFM, Shimadzu SPM-9700	361.80	27
-18.3	**N. A.**	36.5	4.2		153.30	
-8.3	**5.73**	N. A.	N. A.	TEM, JEOL-1230	N. A.	84
-17.7	**5.81**	N. A.	N. A.		N. A.	
-22.0	**5.49**	50.0	N. A.		N. A.	
-25.2	**5.29**	55.0	15.5		852.50	
47.2	**N. A.**	N. A.	N. A.	TEM, Hitachi 7650	N. A.	85
-3.0	**N. A.**	14.5	71		1029.50	
2.2	**N. A.**	15.8	46		728.33	
23.9	**N. A.**	20.0	60		1200.00	
24.0	**N. A.**	15.0	95		1425.00	
33.2	**N. A.**	18.3	84		1540.00	
46.9	**N. A.**	23.3	76		1773.33	
38.1	**N. A.**	16.5	21	AFM, Agilent5500	346.50	86
37.2	**N. A.**	16.0	35.6		569.60	
28.1	**N. A.**	15.0	50.6		759.00	
91.2	**N. A.**	11.0	75.6		831.60	
95.8	**N. A.**	24.0	93.6		2246.40	
44.4	**N. A.**	8.0	71.4	FESEM, SIGMA 300	571.20	87
63.6	**N. A.**	24.0	28.6		686.40	
53.3	**7.60**	8.4	78.5	SEM, Hitachi S4800	659.40	88
59.1	**7.81**	9.3	65.3		607.29	
64.3	**7.99**	11.7	52.6		615.42	
69.8	**8.13**	13.5	43.2		583.20	
78.6	**8.40**	16.8	35.7		599.76	
77.6	**8.19**	16.9	38.3		647.27	
75.2	**N. A.**	3.3	566	SEM, JSM－	1861.84	89

71.4	**N. A.**	5.3	195	7500F, JEOL	1026.32	
59.6	**N. A.**	6.1	135		817.11	
79.8	**N. A.**	7.5	102		765.00	
70.3	**N. A.**	13.4	41.2	SEM, JSM-6700F, JEOL	552.08	90
67.1	**N. A.**	24.0	134.4		3225.60	
40.0	**N. A.**	27.6	132.2		3648.72	
34.1	**N. A.**	48.9	373.3		18254.37	
75.4	**N. A.**	40.0	15	TEM, Tecnai G2 F20 S-Twin	600.00	91
67.9	**8.33**	13.8	32	SPM, Veeco, MultiMode	441.60	95
65.4	**8.04**	14.9	30		447.30	
56.3	**7.90**	17.5	21		367.71	
37.6	**7.10**	20.9	15		313.20	
36.3	**4.49**	24.5	8.6		210.44	
93.4	**8.94**	20.0	19.2	SPM, Veeco, MultiMode	384.00	96
78.3	**8.43**	17.5	23.6		413.00	
88.1	**9.05**	20.0	18.7		374.00	
8.7	**5.32**	45.5	10.2	SEM, ZEISS	464.10	99

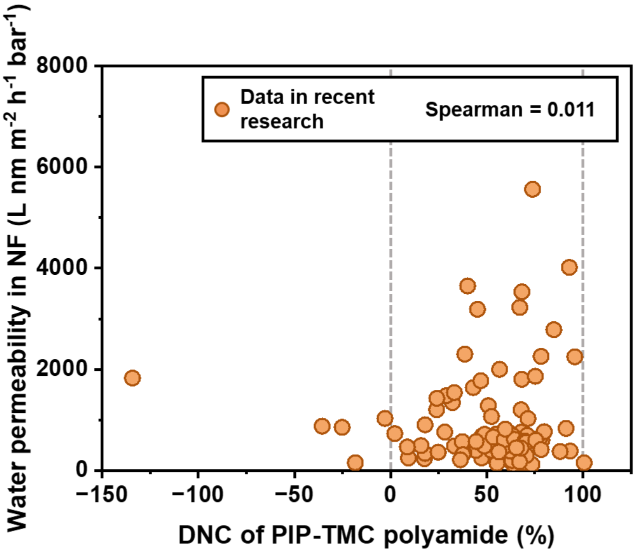

Supplementary Fig. 15. The correlation analysis between DNC and water permeability in NF.

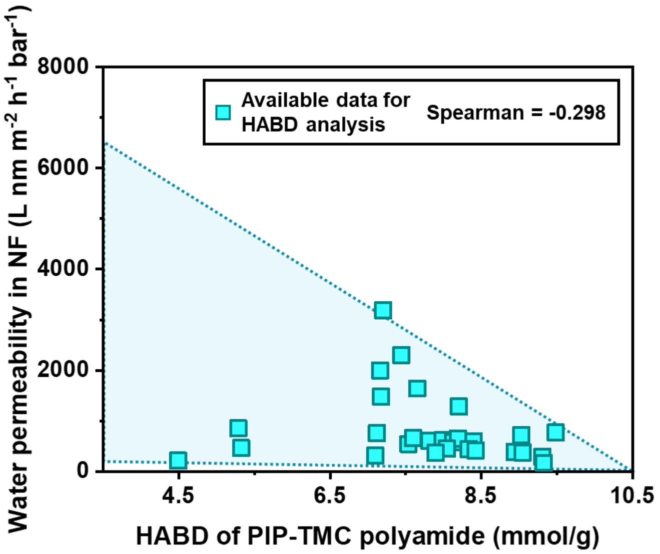

Supplementary Fig. 16. The correlation analysis between HABD and water permeability in NF.

4. HCD and HAD for quantifying terminal groups

We provided two parameters suitable for describing the surface charge of polyamides, named the harmonic carboxyl density (HCD) and the harmonic amino density (HAD) to reflect the contents of carboxyl and amino groups per mass of polyamide, respectively:

$$\mathrm{HCD} = \frac{\alpha}{N_A \mathrm{m}} = \frac{\alpha}{\alpha \mathrm{M}_\alpha + \beta \mathrm{M}_\beta + \gamma \mathrm{M}_\gamma} \tag{4-1}$$

$$\mathrm{HAD} = \frac{\gamma}{N_A \mathrm{m}} = \frac{\gamma}{\alpha \mathrm{M}_\alpha + \beta \mathrm{M}_\beta + \gamma \mathrm{M}_\gamma} \tag{4-2}$$

Through HCD and HAD, we can determine the density of terminal groups on the surface of polyamide and correlate it with the dissociation of polyamide [101]. It can even be mutually confirmed with the terminal group content of polyamide obtained by precision analysis devices such as titration, Quartz Crystal Microbalance or Rutherford Backscattering Spectrometry [102-104].

4.1. Calculation of HCD and HAD in PIP-TMC polyamide

For PIP-TMC polyamide, the ratio of α/β is numerically equivalent to the ratio of carboxyl group to amide bonds since one carboxyl group contains two O1 atom. That is, the ratio of $\frac{\mathrm{O1}}{2}$ to O2. Similarly, the ratio of γ/β is numerically equivalent to the ratio of amino group to amide bonds, which is the ratio of (N1+N3) to N2. N1, N2, and N3 are the number of amino N, amide N, and protonated N of polyamide. Here, amino N are further classified as N1 and N3 for the convenience of peak fitting during XPS data process, which are detailed discussed afterwards.

Thus, HCD and HAD are equal to:

$$\mathrm{HCD} = \frac{\frac{\alpha}{\beta}}{\frac{\alpha}{\beta}\mathrm{M}_\alpha + \mathrm{M}_\beta + \frac{\gamma}{\beta}\mathrm{M}_\gamma} = \frac{\frac{\alpha}{\beta}}{\frac{\mathrm{O1}}{2\mathrm{O2}}\mathrm{M}_\alpha + \mathrm{M}_\beta + \frac{\mathrm{N1+N3}}{\mathrm{N2}}\mathrm{M}_\gamma} \tag{4-3}$$

$$\mathrm{HAD} = \frac{\frac{\gamma}{\beta}}{\frac{\alpha}{\beta}\mathrm{M}_\alpha + \mathrm{M}_\beta + \frac{\gamma}{\beta}\mathrm{M}_\gamma} = \frac{\frac{\gamma}{\beta}}{\frac{\mathrm{O1}}{2\mathrm{O2}}\mathrm{M}_\alpha + \mathrm{M}_\beta + \frac{\mathrm{N1+N3}}{\mathrm{N2}}\mathrm{M}_\gamma} \tag{4-4}$$

4.2. HCD and HAD with ionization of terminal groups

In the actual separation process, it is necessary to consider the hydrolysis of carboxyl and amino groups. Take PIP-TMC polyamide as an example, here are two hydrolysis process in polyamide network:

$$\text{R-COOH} + H_2O \leftrightarrow \text{R-COO}^- + H_3^+O \quad pK_{a1} \approx 2.12$$

$$R_2\text{-NH}_2^+ + H_2O \leftrightarrow \text{R-NH} + H_3^+O \quad pK_{a2} \approx 9.83$$

$$\frac{[H_3^+O][\text{R-COO}^-]}{[\text{R-COOH}]} = 10^{-2.12} \tag{4-5}$$

$$\frac{[H_3^+O][R_2\text{-NH}]}{[R_2\text{-NH}_2^+]} = 10^{-9.83} \tag{4-6}$$

The dissociated carboxyl and amino groups will contribute opposite charge for polyamide, respectively. The total charge of the polyamide nanofilms can be considered to be related to the difference of dissociated carboxyl and amino groups:

$$[\text{R-COO}^-] - [R_2\text{-NH}_2^+] = \frac{10^{-2.12}[\text{R-COOH}]}{[H_3^+O]} - \frac{[H_3^+O][\text{R-NH}]}{10^{-9.83}} \tag{4-7}$$

Considering that the usual nanofiltration process involves separating electrically neutral Na_2SO_4 solutions, pH equals to 7.

$$[\text{R-COO}^-] - [\text{R-NH}_2^+] \propto 10^{4.88}[\text{R-COOH}] - 10^{2.83}[R_2\text{-NH}] \tag{4-8}$$

where $[\text{R-COOH}]$ and $[R_2\text{-NH}]$ are the number of carboxyl and amino groups per unit volume of polyamide, proportional to our HCD and HAD:

$$[\text{R-COO}^-] - [R_2\text{-NH}_2^+] \propto 10^{4.88}\text{HCD} - 10^{2.83}\text{HAD} \approx 10^{4.88}\text{HCD} \tag{4-9}$$

Supplementary Formula 4-9 indicates that the content of surface carboxyl groups (HCD) should theoretically form a positive correlation with the negative charge carried on polyamide nanofilms in pH = 7.

4.3. Zeta potential in NF and HCD

We directly obtained the XPS analysis results from recent researches, including the proportion of C, N, O atoms and the peak splitting results of N1, N2 and N3. We adopted their peak fitting results for N, and excluded the peak fitting results for C or O from the cited works due to their lack of consideration for the cross-peak self-consistency relationship between atoms, such as the equality of N2 and O2. In our calculation of HCD, we directly equated O2 in these works to N2. The remaining portion obtained by subtracting O2 from the total O is assumed to represent O1 of polyamide. The HCD of PIP-TMC polyamide is calculated by Supplementary Formula 4-3 in main text based on the N1, N2, N3 and O1 form corresponding references.

Supplementary Table 9. Summary of Zeta potential, Na_2SO_4 rejection in NF and HCD in PIP-TMC polyamide

N1+N3 (%)	N2 (%)	O1 (%)	HCD (mmol g^{-1})	R (Na_2SO_4) (%)	Zeta potential (mV)	Testing conditions of zeta potential	Ref.
0.33	12.83	2.80	**1.05**	96.5	N. A.	N. A.	63
0.36	12.80	3.35	**1.24**	96.6	N. A.		
2.97	8.43	7.06	**3.00**	99.0	-24	CeO2 + Polysulfone, on Malvern Zetasizer Nano ZS,	64
3.54	8.96	7.25	**2.88**	98.0	-34		
4.09	9.11	4.73	**1.96**	95.8	-42		
0.16	12.90	3.83	**1.40**	97.2	N. A.	N. A.	72
0.07	11.16	8.55	**3.13**	99.2	N. A.		
0.30	11.65	7.34	**2.66**	97.8	N. A.		
0.16	12.90	4.03	**1.47**	98.4	N. A.		
2.25	8.22	7.64	**3.33**	95.0	-8	Alginate Hydrogel + Polyethersulfone, on SurPASS, Anton Paar GmbH, Graz	75
2.06	8.20	6.65	**3.02**	97.0	-35		
2.36	8.18	7.31	**3.22**	96.0	-35		
2.21	9.31	6.73	**2.77**	93.5	-30		
0.93	16.76	11.67	**2.86**	97.5	-35		
N. A.	N. A.	N. A.	**3.79** [a]	92.0	-40	EVOH + PET, on Austrian Anton Paar surpass	78
N. A.	N. A.	N. A.	**3.31** [a]	95.5	-52		
0.31	11.58	4.79	**1.87**	94.0	-24	Gelatin + PSf, on SurPASSTM3, Anton-Paar GmbH	79
N. A.	N. A.	N. A.	**N. A.**	98.5	-28		
0.40	11.88	3.02	**1.20**	98.5	-28		
N. A.	N. A.	N. A.	**N. A.**	98.5	-48		
N. A.	N. A.	N. A.	**N. A.**	98.5	-50		
0.30	5.39	1.54	**1.33**	91.3	N. A.	N. A.	80
0.38	9.48	2.81	**1.38**	96.4	N. A.		

2.40	7.10	13.14	**5.31**	N. A.	N. A.	N. A.	84
1.95	7.55	14.21	**5.47**	N. A.	N. A.		
2.11	6.78	14.29	**5.78**	94.0	N. A.		
2.30	6.71	15.21	**5.99**	91.0	N. A.		
N. A.	N. A.	N. A.	**3.96** [a]	98.4	-38.83	Amino functionalized PSf, on SurPASSTM3	88
N. A.	N. A.	N. A.	**3.65** [a]	99.2	-34.71		
N. A.	N. A.	N. A.	**3.31** [a]	99.5	-27.84		
N. A.	N. A.	N. A.	**3.06** [a]	99.5	-24.12		
N. A.	N. A.	N. A.	**2.54** [a]	99.2	-15.41		
N. A.	N. A.	N. A.	**2.78** [a]	99.0	-12.54		
2.05	10.74	5.12	**1.98**	99.7	-22	PES, on SurPASS Anton Paar, GmbH	95
2.33	10.12	5.58	**2.22**	99.6	-24		
2.20	10.09	6.39	**2.50**	99.5	-25		
2.58	9.08	8.71	**3.40**	99.4	-25		
5.86	5.22	11.83	**5.08**	97.8	-28		
2.22	11.69	2.85	**1.09**	99.6	N. A.	N. A.	96
2.28	10.54	4.28	**1.71**	98.8	N. A.		
1.82	11.77	2.94	**1.13**	98.0	N. A.		
3.21	7.02	9.63	**4.21**	N. A.	N. A.	N. A.	98
2.82	6.41	13.82	**5.68**	95.2	N. A.		
3.77	6.37	11.77	**4.99**	97.0	N. A.		
2.91	6.93	8.47	**3.91**	98.0	N. A.		
3.58	6.26	12.19	**5.18**	98.6	N. A.	N. A.	99

a: This work directly provided the proportion of carboxyl group, amide bond, and amino group.

4.4. Correlation between Zeta potential in NF and HCD

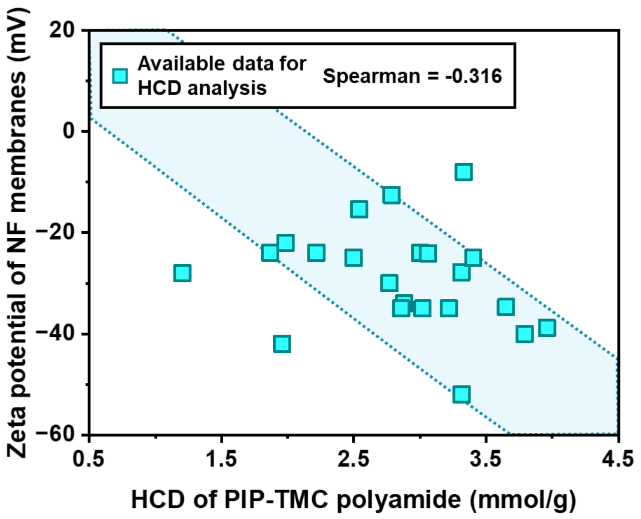

Supplementary Fig. 17. The correlation analysis between HCD with Zeta potential in NF.

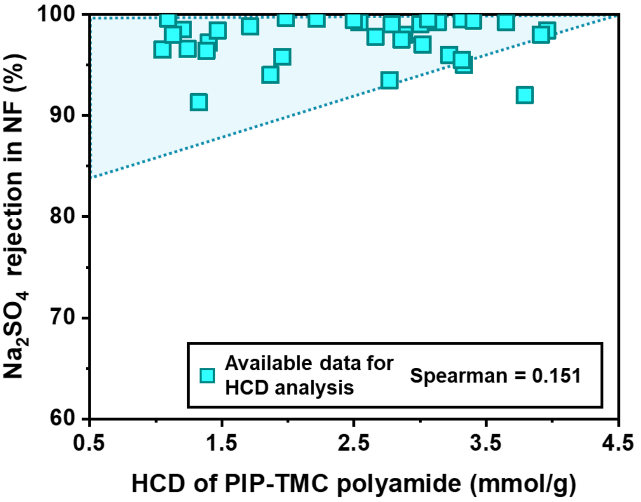

Supplementary Fig. 18. The correlation analysis between HCD with Na_2SO_4 rejection in NF.

5. XPS test conditions

5.1. XPS test conditions in reference for obtaining XPS data

Supplementary Table 10. XPS test conditions of RO membranes in Fig. 3 in main text.

Sample conditioning	Instruments	Vacuum level	Area of analysis	X-ray type and energy	X-ray exposure time	Data analysis	Ref.
TFC membrane	Axis Ultra DLD, Kratos Analytical	N. A.	N. A.	N. A.	N. A.	CasaXPS software	18
TFC membrane	AXIS Supra	N. A.	300 × 700 μm	Al Kα (1486.6 eV)	N. A.	CasaXPS software	19
TFC membrane	Thermo Scientific49 ESCALAB 250Xi,	N. A.	N. A.	N. A.	N. A.	N. A.	20
TFC membrane, rinsed 3 times with ultrapure water followed by freeze-drying	Kratos AXIS Supra (UK) system	N. A.	N. A.	Al Kα	N. A.	N. A.	21
TFC membrane	K-alpha	N. A.	N. A.	Al Kα	N. A.	N. A.	22
TFC membrane	X-tool spectrometer, ULVAC-PHI	N. A.	N. A.	N. A.	N. A.	N. A.	23

TFC membrane	Kratos Inc., AXIS–His	N. A.	N. A.	Al (1486.6eV)	N. A.	N. A.	[24]
TFC membrane	JPS-9010 MC, JEOL	N. A.	N. A.	N. A.	N. A.	N. A.	[25]
TFC membrane	ESCALAB 250 XI	N. A.	N. A.	N. A.	N. A.	N. A.	[26]
TFC membrane	Quanta 200 spectrometer	N. A.	N. A.	N. A.	N. A.	N. A.	[27]
EDX	N. A.	N. A.	N. A.	N. A.	N. A.	N. A.	[28]
TFC membrane	VG Multilab 2000, Thermo VG Scientific	N. A.	N. A.	Al Kα (1486.6 eV)	N. A.	N. A.	[29]
TFC membrane	Axis Ultra DLD, Kratos Analytical	N. A.	N. A.	N. A.	N. A.	N. A.	[30]
TFC membrane	Escalab 250Xi, Thermo Fisher Scientific	N. A.	N. A.	N. A.	N. A.	N. A.	[31]
EDX	N. A.	N. A.	N. A.	N. A.	N. A.	N. A.	[32]
TFC membrane	Thermal Scientific K-Alpha	N. A.	N. A.	N. A.	N. A.	N. A.	[33]
TFC membrane	ULVAC PHI X-tool	N. A.	N. A.	N. A.	N. A.	N. A.	[34]
TFC membrane	Shimadzu, AXIS Ultra DLD	N. A.	N. A.	N. A.	N. A.	N. A.	[35]

TFC membrane	N. A.	6×10^{-10} Torr	N. A.	Al Kα (1486.6 eV)	N. A.	N. A.	36
TFC membrane	Quanta 200 spectrometer, FEI.	N. A.	N. A.	N. A.	N. A.	N. A.	37
TFC membrane	ESCALAB 250Xi	N. A.	N. A.	N. A.	N. A.	N. A.	38
TFC membrane	ESCALAB 250 XI	N. A.	N. A.	N. A.	N. A.	N. A.	39
TFC membrane	X-tool spectrometer, ULVAC-PHI	N. A.	N. A.	N. A.	N. A.	N. A.	40
Self-standing PA nanofilm	PHI-5000	N. A.	N. A.	Al Kα (1.49 keV)	N. A.	N. A.	41
TFC membrane	Kratos AXIS ULTRA, UK	N. A.	N. A.	N. A.	N. A.	N. A.	42
TFC membrane	Thermo escalab 250Xi	N. A.	N. A.	Al Kα (1486.6 eV)	N. A.	N. A.	43
TFC membrane	PHI-1600	N. A.	N. A.	N. A.	N. A.	N. A.	44
TFC membrane	Thermo escalab 250Xi, USA	N. A.	N. A.	N. A.	N. A.	N. A.	45
TFC membrane	Thermo Electron, K-Alpha	N. A.	N. A.	N. A.	N. A.	N. A.	46

TFC membrane	Thermo Fisher Scientific Co., US	N. A.	N. A.	N. A.	N. A.	N. A.	[47]
TFC membrane	PHI-5000	N. A.	N. A.	Al Kα (1486.6 eV)	N. A.	N. A.	[48]
TFC membrane	Surface Science Instruments model ESCALAB250	N. A.	N. A.	Al Kα (1486.6 eV)	N. A.	N. A.	[49]
TFC membrane	Kratos AXIS ULTRA, UK	N. A.	N. A.	Al Kα (1486.6 eV)	N. A.	N. A.	[50]
N. A.	N. A.	N. A.	N. A.	N. A.	N. A.	N. A.	[51]
TFC membrane, storage in deionized water and completely dried under vacuum before XPS	PHI-1600	N. A.	N. A.	Mg Kα	N. A.	PHI-MATLAB software package	[52]
Self-standing PA nanofilm loaded on gold-plated silicon wafers	Escalab250Xi, ThermoFisher	N. A.	N. A.	Al Kα (1486.6 eV)	N. A.	N. A.	[9]
TFC membrane	PHI-1600	N. A.	N. A.	Al Kα (1486.6 eV)	N. A.	N. A.	[53]

Self-standing PA nanofilm loaded on silicon wafers	Thermo Scientific K-Alpha	N. A.	N. A.	Al Kα (1486.6 eV)	N. A.	N. A.	54
Self-standing PA nanofilm loaded on silicon wafers	Thermo Scientific K-Alpha	N. A.	N. A.	Al Kα (1486.6 eV)	N. A.	N. A.	55
TFC membrane	K-Alpha, Thermo Fisher Scientific	N. A.	N. A.	N. A.	N. A.	N. A.	56
TFC membrane	ThermoFisher K-alpha	N. A.	N. A.	Al Kα (1486.6 eV)	N. A.	N. A.	57
TFC membranes, exten sively rinsed and soaked in MilliQ water for 24 h before dried in a vacuum.	SSI S-Probe Monochromatized XPS Spectrometer	N. A.	250 μm × 1000 μm	Al Kα (1486.6 eV)	N. A.	N. A.	60

Supplementary Table 11. XPS test conditions of NF membranes in Fig. 4 in main text.

Sample conditioning	Instruments	Vacuum level	Area of analysis	X-ray type and energy	X-ray exposure time	Data analysis	Ref.
TFC membranes	ESCALAB 250	N. A.	N. A.	N. A.	N. A.	N. A.	62

TFC membranes	Thermal Fisher Scientific ESCALAB 250 Xi)	N. A.	N. A.	N. A.	N. A.	XPSPEAK41 software	63
TFC membranes	XRF-1800	N. A.	N. A.	N. A.	N. A.	N. A.	64
TFC membranes	K-alpha, Thermo Fisher, USA	N. A.	N. A.	N. A.	N. A.	N. A.	65
TFC membranes	K-alpha, Thermo Fisher Scientific	N. A.	N. A.	Al Kα (1486.6 eV)	N. A.	N. A.	66
TFC membranes	Kratos Analytical-A	N. A.	N. A.	N. A.	N. A.	N. A.	67
TFC membranes	Thermal Fisher Scientific ESCALAB	N. A.	N. A.	N. A.	N. A.	XPSPEAK software	68
TFC membranes	Axis Ultra DLD	N. A.	N. A.	N. A.	N. A.	N. A.	69
TFC membranes	AXIS UltraDLD	N. A.	N. A.	N. A.	N. A.	N. A.	70
N. A.	ESCALAB250Xi	N. A.	N. A.	N. A.	N. A.	N. A.	71
N. A.	N. A.	N. A.	N. A.	N. A.	N. A.	N. A.	72
TFC membranes	JPS-9010 MC	N. A.	N. A.	N. A.	N. A.	N. A.	73
TFC membranes	Kratos Axis Ultra DLD, UK	N. A.	N. A.	Al Kα (1486.6 eV)	N. A.	N. A.	74

TFC membranes	Thermo Scientific K-Alpha	N. A.	N. A.	Al Kα (1486.6 eV)	N. A.	N. A.	75
N. A.	N. A.	N. A.	N. A.	N. A.	N. A.	N. A.	76
TFC membranes	ESCALAB 250	N. A.	N. A.	N. A.	N. A.	N. A.	77
TFC membranes, samples were placed under the infrared lamp for 1 h.	Thermo Kalpha	N. A.	N. A.	N. A.	N. A.	N. A.	78
TFC membranes	Quanta200 spectrometer	N. A.	N. A.	N. A.	N. A.	N. A.	79
TFC membranes	N. A.	N. A.	N. A.	N. A.	N. A.	N. A.	80
TFC membranes	Thermo Scientific MultiLab 2000	N. A.	specific spot size of 650 lm	Al Kα (1486.6 eV)	N. A.	N. A.	81
TFC membranes	Thermo Fisher S4 Scientific Escalab 250Xi	N. A.	N. A.	Al Kα (1486.6 eV)	N. A.	N. A.	82
TFC membranes	Quanta 200 spectrometer	N. A.	N. A.	N. A.	N. A.	N. A.	27
TFC membranes	PerkinElmer	N. A.	N. A.	Al Kα (1486.6 eV)	N. A.	N. A.	83

TFC membranes	AXIS Supra	N. A.	N. A.	Al Kα (1486.6 eV)	N. A.	CasaXPS software	84
TFC membranes	Thermo Scientific	N. A.	N. A.	N. A.	N. A.	N. A.	85
TFC membranes	ESCALAB250Xi	N. A.	N. A.	N. A.	N. A.	N. A.	86
TFC membranes	Quanta 200	N. A.	N. A.	N. A.	N. A.	N. A.	87
TFC membranes	Quanta 200	N. A.	N. A.	N. A.	N. A.	N. A.	88
TFC membranes	Kratos AXIS Ultra DLD	N. A.	N. A.	N. A.	N. A.	The Shirley-type back ground and the Gaussian-Lorentz peak deconvolution were used for fitting the high-resolution C 1*s* spectra	89
TFC membranes	K-alpha, Thermo Fisher	N. A.	N. A.	N. A.	N. A.	N. A.	90
TFC membranes	Thermo Fisher Scientific ESCALAB 250Xi XPS	N. A.	N. A.	N. A.	N. A.	N. A.	91
TFC membranes, freeze dried before test	K-alpha, Thermo Fisher	N. A.	N. A.	N. A.	N. A.	N. A.	92
TFC membranes	PerkinElmer 5300	N. A.	N. A.	Al Kα (1486.6 eV)	N. A.	N. A.	93

TFC membranes	K-alpha, Thermo Fisher Scientific	N. A.	N. A.	N. A.	N. A.	N. A.	[94]
TFC membranes	ThermoScientific, K Alpha+	N. A.	N. A.	Al Kα (1486.6 eV)	N. A.	N. A.	[95]
TFC membranes	ThermoScientific, K-Alpha+	N. A.	N. A.	Al Kα (1486.6 eV)	N. A.	N. A.	[96]
TFC membranes	PerkinElmer	N. A.	N. A.	Al Kα (1486.6 eV)	N. A.	N. A.	[97]
TFC membranes	PerkinElmer	N. A.	N. A.	Al Kα (1486.6 eV)	N. A.	N. A.	[98]
TFC membranes	Thermo-Fisher	N. A.	N. A.	N. A.	N. A.	N. A.	[99]
TFC membranes, exten sively rinsed and soaked in MilliQ water for 24 h before dried in a vacuum.	SSI S-Probe Monochromatized XPS Spectrometer	N. A.	250 μm × 1000 μm	Al Kα (1486.6 eV)	N. A.	N. A.	[60]

5.2. Recommended XPS testing conditions

In order to improve the standardization of XPS testing as well as the accuracy of the obtained peak fitting results and calculated HABD, we referred to the standards of International Organization for Standardization (ISO 20579), American Society for Testing Materials (E1078), and National Standard of the People's Republic of China (GB/T SJT10458-1993) and related researches [16,105,106] to provide a series of recommendations regarding sample preparation and testing conditions for analyzing the surface of polyamides. We have summarized the key points as follows:

Sample preparation: For the conventional polyamide thin-film composite membranes, cleaning the solvents and unreacted monomers is crucial. We suggest choosing to soak and clean with ethanol for more than 24 hours. For the free-standing polyamide nanofilms developed in recent years, it can be considered to use silicon wafers or gold-plated silicon wafers to load samples for testing to avoid interference from foreign elements. After drying, the polyamide nanofilm can firmly adhere to the surface of the silicon wafer and should also be cleaned before testing.

Storage time: Minimize the storage time of test samples as much as possible. If storage is necessary, water can be considered to seal the samples, but drying should be carried out before testing. If possible, it is recommended to use rare gas to protect the sample. In our method, it is not recommended to protect the sample with N_2, because the subsequent analysis mainly uses N as the calibration.

Vacuum level: The vacuum level during XPS test should be below than 10^{-7} mbar.

X-ray beam spots: We recommend using larger X-ray beam spots (more than 200 μm) to observe polyamide in order to obtain results that better reflect the overall element proportion of polyamide. Readers can also perform mapping imaging with small X-ray beam spots and take the average of multiple test points on a large scale.

X-ray source: A stronger X-ray source is not recommended, as it may cause damage to the C-chain polymer [107,108]. Mg Kα (1253.6 eV with natural linewidth of 0.7 eV) and Al Kα (1486.6 eV with natural linewidth of 0.9 eV) can obtain information about the surface of polyamide at around 7~10 nm, which is acceptable. Moreover, the reported polyamide nanofilm has become

thinner in recent years, and the comprehensiveness of Mg Kα or Al Kα is acceptable.

X-ray exposure time: X-rays will also cause certain damage to polyamide with exposure time prolong [56,109]. Considering that polyamides typically require testing full spectrum and fine spectrum of C, N, and O in XPS, the total XPS irradiation time should not exceed 5 min.

We expect researchers to refer to relevant standards and researches before conducting XPS test. Following above key points can help to improve the accuracy of peak fitting result and to assist in the analysis of RO and NF materials along with the proposed HABD.

6. HABD for new polyamide membranes

Our segmentation method is based on the bond characteristics of polymers, so it is suitable for the segmentation of polyamide, polyurea, polyester, COF and kinds of crosslinking networks [110-113]. This segmentation method needs to divide the corresponding monomers equally according to their functionality, each structure cell should contain and only contain one reactive group. Since this segmentation method is mainly used for XPS, it is not considered whether the H atom can be evenly divided. Monomers that can be segmented in this way will be suitable for this method. According to these principles, some available monomers are shown in Supplementary Table 12. We demonstrated some polyamides as examples. The polyamides synthesized by other monomers can follow the ideas provided in this work to solve the calculation formulas of HABD.

Supplementary Table 12. Monomers of polymers which our segmentation method and HABD are suited for.

Monomer structure	Monomer	Membrane
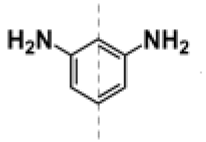	MPD, m-phenylenediamine	Reverse osmosis[114], Organic solvent nanofiltration[13], Nanofiltration[115], Gas separation[9].
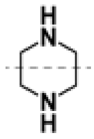	PIP, piperazine	Organic solvent nanofiltration[13], Nanofiltration[116], Gas separation[9].
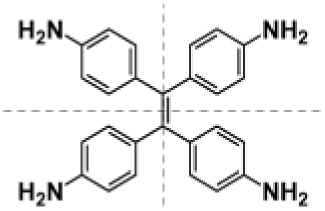	TAPE, tetrakis (4-aminophenyl) ethene	Organic solvent nanofiltration, Gas separation[9].

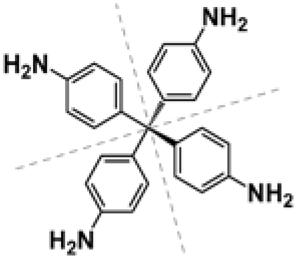	TAM, tetrakis (4-aminophenyl) methane	Organic solvent nanofiltration, Gas separation[9].
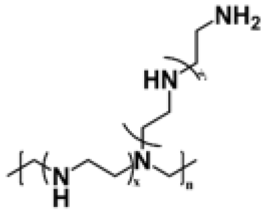	PEI, polyethylenimine	Nanofiltration[117,118].
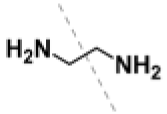	EDA, ethylenediamine	Nanofiltration[119].
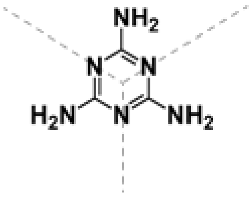	Melamine	Nanofiltration[120].
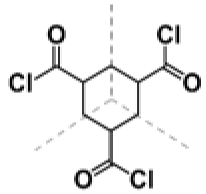	HTC, cyclohexane-1,3,5-tricarbonyl chloride	Reverse osmosis[121], Nanofiltration[122].
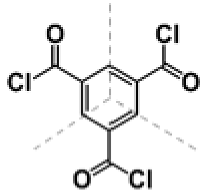	TMC, trimesoyl chloride	Reverse osmosis[123], Organic solvent nanofiltration[13], Nanofiltration[123], Gas separation[9].
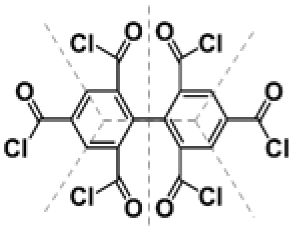	BHAC, 2,2`,4,4`,6,6`-biphenyl hexaacyl chloride	Reverse osmosis[124], Nanofiltration[125].
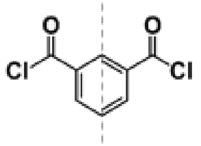	IPC, isophthaloyal chloride	Nanofiltration[126].

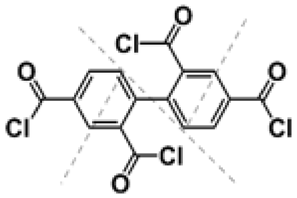	op-BTEC, 2,2`,5,5`-biphenyl tetraacyl chloride	Reverse osmosis[127].
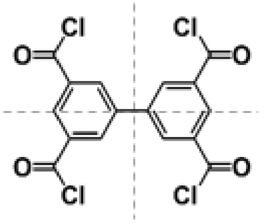	mm-BTEC, 3,3`,5,5`-biphenyl tetraacyl chloride	Reverse osmosis[128], Nanofiltration[129].
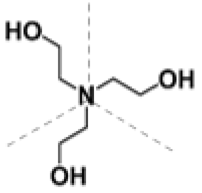	TEOA, triethanolamine	Nanofiltration[130].
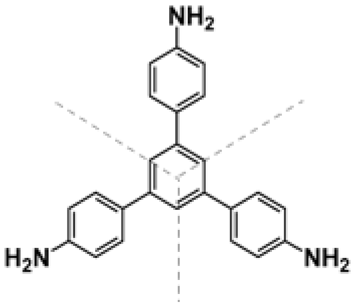	TAPB, 1,3,5-tris(4-aminophenyl)-benzene	Semiconductor[131]
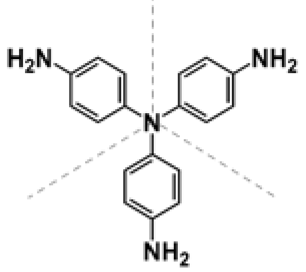	TAPA, tris (4-aminophenyl)amine	Semiconductor[132]
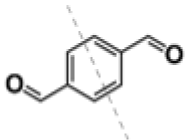	TPA, terephthalaldehyde	Nanofiltration[133].
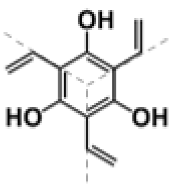	TFP, 1,3,5-triformylphloroglucinol	Organic solvent nanofiltration[134]

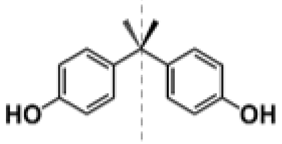	BPA, bisphenol A	Nanofiltration[135].
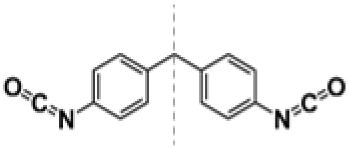	MDI, 1-isocyanato-4-[(4-isocyanatophenyl) methyl] benzene	Microcapsules[136]
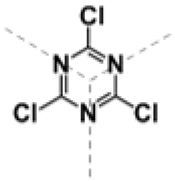	CC, cyanuric chloride	Nanofiltration[137].

6.1. TAM-TMC polyamide

Tetra (4-aminophenyl) methane (TAM) is a tetrahedral tetrafunctional amine monomer. The polyamide synthesized by the reaction of TAM and TMC (TAM-TMC polyamide) has a complex topological structure, but it can still be divided into three types of structure cells (Supplementary Fig. 19). The statistical results of various atoms are shown in Supplementary Table 13.

For TAM-TMC polyamide, an amino structure cell γ only contains one N1 or N3, while an amide bond structure cell β only contains one N2:

$$\frac{\mathrm{N1+N3}}{\mathrm{N2}} = \frac{\gamma}{\beta} \tag{6-1}$$

A carboxyl structure cell α contains two O1, while an amide bond structure cell β only contains one O2:

$$\frac{\mathrm{O1}}{\mathrm{O2}} = \frac{2\alpha}{\beta} \tag{6-2}$$

HABD equal to:

$$\mathrm{HABD} = \frac{1}{\frac{\mathrm{O1}}{2\mathrm{O2}}\mathrm{M}_{\alpha}+\mathrm{M}_{\beta}+\frac{\mathrm{N1+N3}}{\mathrm{N2}}\mathrm{M}_{\gamma}} \tag{6-3}$$

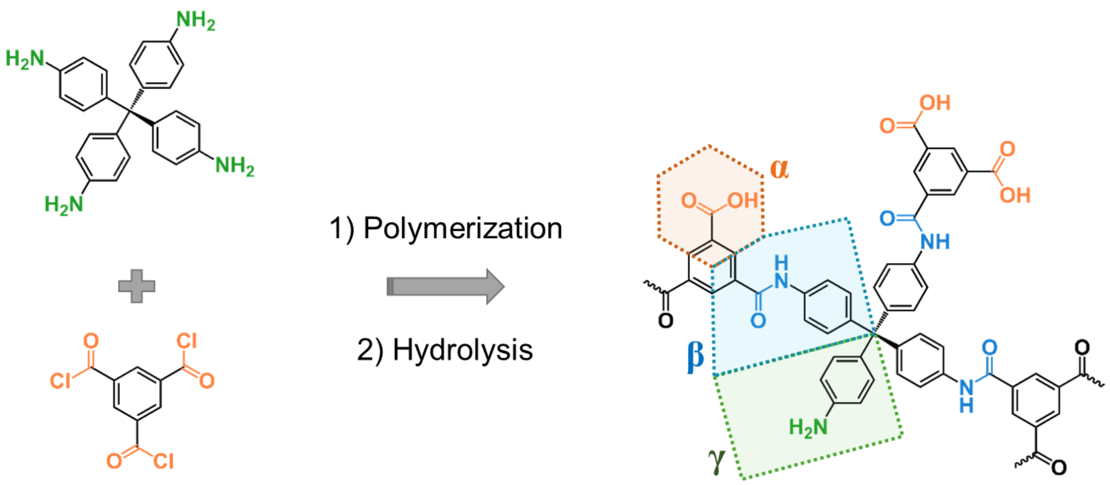

Supplementary Fig. 19. Schematic of the segmentation and synthesis process of TAM-TMC polyamide from tetra (4-aminophenyl) methane (TAM) and trimesoyl chloride (TMC).

Supplementary Table 13. Classification and number of various atoms in TAM-TMC polyamide

Type	Located Group	α	β	γ
C1	C-COOH	1	0	0
C2	C-CONH-	0	1	0
C3	C-NH_2, C-NHCO-	0	1	1
C4	C-CONH, C-COOH	1	1	0
C5	C-H, C-C, C=C	1	6.25	5.25
	**Total C**	**3**	**9.25**	**6.25**
O1	-COOH	2	0	0
O2	CONH	0	1	0
	**Total O**	**2**	**1**	**0**
N1	R-NH_2	0	0	1
N3	R-$N^{+}H_3$			
N2	CONH	0	1	0
	**Total N**	**0**	**1**	**1**

6.2. TAPE-TMC polyamide

Tetra (4-aminophenyl) ethene (TAPE) is a tetrafunctional monomer. When the TAPE-TMC polyamide is segmented (Supplementary Fig. 20), The double bond in the center of TAPE will be quartered, and each structure cell γ will obtain an average of 0.5 C atoms. The number of atoms in other structure cells is shown in Supplementary Table 14.

For TAPE-TMC polyamide, an amino structure cell γ only contains one N1 or N3, while an amide bond structure cell β only contains one N2:

$$\frac{N1+N3}{N2} = \frac{\gamma}{\beta} \tag{6-4}$$

A carboxyl structure cell α contains two O1, while an amide bond structure cell β only contains one O2:

$$\frac{O1}{O2} = \frac{2\alpha}{\beta} \tag{6-5}$$

HABD equal to:

$$\mathrm{HABD} = \frac{1}{\frac{O1}{2O2}M_{\alpha}+M_{\beta}+\frac{N1+N3}{N2}M_{\gamma}} \tag{6-6}$$

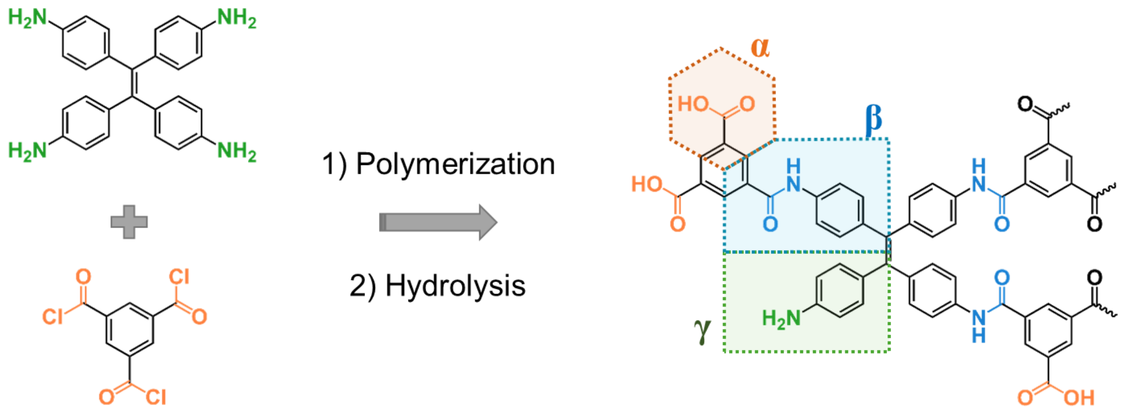

Supplementary Fig. 20. Schematic of the segmentation and synthesis process of TAPE-TMC polyamide from tetra (4-aminophenyl) ethene (TAPE) and trimesoyl chloride (TMC).

Supplementary Table 14. Classification and number of various atoms in TAPE-TMC polyamide.

Type	Located Group	α	β	γ
C1	C-COOH	1	0	0
C2	C-CONH-	0	1	0
C3	C-NH_2, C-NHCO-	0	1	1
C4	C-CONH, C-COOH	1	1	0
C5	C-H, C-C, C=C	1	6.5	5.5
	**Total C**	**3**	**9.5**	**6.5**
O1	-COOH	2	0	0
O2	CONH	0	1	0
	**Total O**	**2**	**1**	**0**
N1	R-NH_2	0	0	1
N3	R-$N^{+}H_3$			
N2	CONH	0	1	0
	**Total N**	**0**	**1**	**1**

6.3. TAPE-BTEC polyamide

Polyamides formed from tetrafunctional amines and tetrafunctional acyl chloride have extremely complex topological structures. There are few studies on the synthesis of such polyamides. Therefore, we only take tetra (4-aminophenyl) ethene (TAPE) and 2,2’,4,4’-biphenyl tetradecyl chloride (BTEC) as an example (Supplementary Fig. 21). Such polyamides still satisfy the atomic identity based on chemical bonds. Because there are two acyl chloride groups on an average single benzene ring on the BTEC monomer, and basically distributed in pairs. We can divide half a benzene ring and one carboxyl group as the structure cell α from the fabricated and hydrolyzed polyamide (The number of H atoms cannot be divided equally, but it does not affect the calculation results). The segmentation of structure cell β is similar to the previous text. The number of atoms in structure cells is shown in Supplementary Table 15.

For TAPE-BTEC polyamide, an amino structure cell γ only contains one N1 or N3, while

an amide bond structure cell β only contains one N2:

$$\frac{N1+N3}{N2}=\frac{\gamma}{\beta} \quad (6\text{-}7)$$

A carboxyl structure cell α contains two O1, while an amide bond structure cell β only contains one O2:

$$\frac{O1}{O2}=\frac{2\alpha}{\beta} \quad (6\text{-}8)$$

HABD equal to:

$$\mathrm{HABD}=\frac{1}{\frac{O1}{2O2}M_{\alpha}+M_{\beta}+\frac{N1+N3}{N2}M_{\gamma}} \quad (6\text{-}9)$$

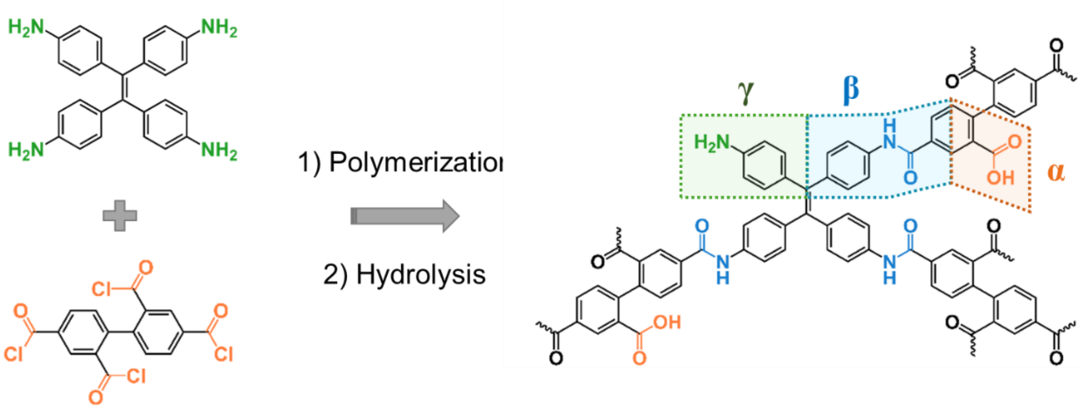

Supplementary Fig. 21. Schematic of the segmentation and synthesis process of TAPE-BTEC polyamide from tetra (4-aminophenyl) ethene (TAPE) and 2,2',4,4'-biphenyl tetradecyl chloride (BTEC).

Supplementary Table 15. Classification and number of various atoms in TAPE-BTEC polyamide

Type	Located Group	α	β	γ
C1	C-COOH	1	0	0
C2	C-CONH-	0	1	0
C3	C-NH_2, C-NHCO-	0	1	1
C4	C-CONH, C-COOH	1	1	0
C5	C-H, C-C, C=C	2	7.5	5.5
	**Total C**	**4**	**10.5**	**6.5**

O1	-C$\underline{OO}$H	2	0	0
O2	C$\underline{O}$NH	0	1	0
	**Total O**	**2**	**1**	**0**
N1<br>N3	R-$\underline{N}H_2$<br>R-$\underline{N}^{+}H_3$	0	0	1
N2	CO$\underline{N}$H	0	1	0
	**Total N**	**0**	**1**	**1**

6.4. PEI-TMC polyamide

Poly (ethylene imine) (PEI) is a very special amine monomer, which has no symmetry but periodicity. It is generally believed that in each repeating unit of PEI, only the terminal amino group has strong reactivity. Therefore, we believe that only this group participates in the reaction, and divides the repeat unit of PEI into structure cells γ (Supplementary Fig. 22). The PEI monomer is a mixture of branched PEIs with different molecular weights. We can only use the average results obtained from molecular weight and structure characterization tests as x and y in each repeating unit, and take this as a reference to obtain a fuzzy value of the abundance of structure cells. The y here is related to the side chain length of PEI; x is related to the length of the main chain in the repeating unit. The number of atoms in other structure cells is shown in Supplementary Table 16.

For PEI-TMC polyamide, an amino structure cell γ contains x+y+2 N1 or N3, while an amide bond structure cell β contains one N2 and x+y+1 N1 or N3:

$$\frac{N1+N3}{N2} = \frac{(x+y+2)\gamma+(x+y+1)\beta}{\beta} \quad (6\text{-}10)$$

Means:

$$\frac{\gamma}{\beta} = \frac{N1+N3-(x+y+1)N2}{(x+y+2)N2} \quad (6\text{-}11)$$

An carboxyl structure cell α contains two O1, while an amide bond structure cell β only contains one O2:

$$\frac{O1}{O2} = \frac{2\alpha}{\beta} \quad (6\text{-}12)$$

HABD equal to:

$$\text{HABD} = \frac{1}{\frac{O1}{2O2}M_{\alpha}+M_{\beta}+\frac{N1+N3-(x+y+1)N2}{N2(x+y+2)}M_{\gamma}} \tag{6-13}$$

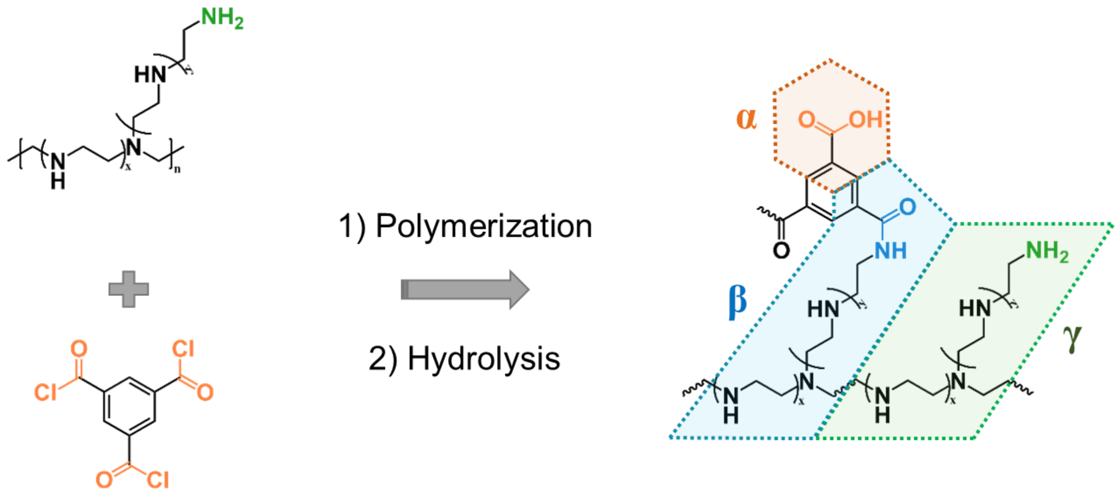

Supplementary Fig. 22. Schematic of the segmentation and synthesis process of PEI-TMC polyamide from poly (ethylene imine) (PEI) and trimesoyl chloride (TMC).

Supplementary Table 16. Classification and number of various atoms in PEI-TMC polyamide

Type	Located Group	α	β	γ
C1	C-COOH	1	0	0
C2	C-CONH-	0	1	0
C3	C-N-	0	2x+2y+4	2x+2y+4
C4	C-CONH, C-COOH	1	1	0
C5	C-H, C-C, C=C	1	1	0
	**Total C**	**3**	**2x+2y+7**	**2x+2y+4**
O1	-COOH	2	0	0
O2	-CONH	0	1	0
	**Total O**	**2**	**1**	**0**
N1	R_2-N-	0	x+y+1	x+y+2
N2	R_2-N^{+}H-			
N2	-CONH	0	1	0
	**Total N**	**0**	**x+y+2**	**x+y+2**

7. Supplementary References and Notes

1 Gaussian 16 Rev. C.01 (Wallingford, CT, 2016).

2 Grimme, S., Antony, J., Ehrlich, S. & Krieg, H. A consistent and accurate ab initio parametrization of density functional dispersion correction (DFT-D) for the 94 elements H-Pu. *J. Chem. Phys.* **132**, 154104 (2010).

3 Grimme, S., Ehrlich, S. & Goerigk, L. Effect of the damping function in dispersion corrected density functional theory. *J. Comput. Chem.* **32**, 1456-1465 (2011).

4 Weigend, F. & Ahlrichs, R. Balanced basis sets of split valence, triple zeta valence and quadruple zeta valence quality for H to Rn: Design and assessment of accuracy. *Phys. Chem. Chem. Phys.* **7**, 3297-3305 (2005).

5 Martínez, L., Andrade, R., Birgin, E. G. & Martínez, J. M. PACKMOL: A package for building initial configurations for molecular dynamics simulations. *J. Comput. Chem.* **30**, 2157-2164 (2009).

6 Abbott, L. J., Hart, K. E. & Colina, C. M. Polymatic: a generalized simulated polymerization algorithm for amorphous polymers. *Theor. Chem. Acc.* **132**, 1334 (2013).

7 Jimenez-Solomon, M. F., Song, Q., Jelfs, K. E., Munoz-Ibanez, M. & Livingston, A. G. Polymer nanofilms with enhanced microporosity by interfacial polymerization. *Nat. Mater.* **15**, 760-767 (2016).

8 Lu, T. & Chen, F. Multiwfn: A multifunctional wavefunction analyzer. *J. Comput. Chem.* **33**, 580-592 (2012).

9 Liu, C. *et al.* Interfacial Polymerization at the Alkane/Ionic Liquid Interface. *Angew. Chem., Int. Ed.* **60**, 14636-14643 (2021).

10 Lu, T. & Chen, F. Quantitative analysis of molecular surface based on improved Marching Tetrahedra algorithm. *J. Mol. Graph. Model.* **38**, 314-323 (2012).

11 Humphrey, W., Dalke, A. & Schulten, K. VMD: Visual molecular dynamics. *J. Mol. Graph.* **14**, 33-38 (1996).

12 Ariza, M. J., Benavente, J., Rodriguez-Castellon, E. & Palacio, L. Effect of hydration of polyamide membranes on the surface electrokinetic parameters: Surface characterization by X-ray photoelectronic spectroscopy and atomic force microscopy. *J. Colloid Interface Sci.* **247**, 149-158 (2002).

13 Karan, S., Jiang, Z. W. & Livingston, A. G. Sub-10 nm polyamide nanofilms with ultrafast solvent transport for molecular separation. *Science* **348**, 1347-1351 (2015).

14 Do, V. T., Tang, C. Y., Reinhard, M. & Leckie, J. O. Degradation of polyamide nanofiltration and reverse osmosis membranes by hypochlorite. *Environ. Sci. Technol.* **46**, 852-859 (2012).

15 Major, G. H., Fernandez, V., Fairley, N., Smith, E. F. & Linford, M. R. Guide to XPS data analysis: Applying appropriate constraints to synthetic peaks in XPS peak fitting. *J. Vac. Sci. Technol. A* **40**, 063201 (2022).

16 Major, G. H. *et al.* Practical guide for curve fitting in x-ray photoelectron spectroscopy. *J. Vac. Sci. Technol. A: Vacuum, Surfaces, and Films* **38**, 061203 (2020).

17 Greczynski, G. & Hultman, L. Self-consistent modelling of X-ray photoelectron spectra from air-exposed polycrystalline TiN thin films. *Appl. Surf. Sci.* **387**, 294-300 (2016).

18 Xu, R. Z. *et al.* Influences of support layer hydrophilicity on morphology and performances of polyamide thin-film composite membrane. *Sep. Purif. Technol.* **281**,

119884 (2022).
19 Lim, Y. J. *et al.* Unraveling the role of support membrane chemistry and pore properties on the formation of thin-film composite polyamide membranes. *J. Membr. Sci.* **640**, 119805 (2021).
20 Huang, J. J. *et al.* Impacts of hydrophobic, hydrophilic, superhydrophobic and superhydrophilic nanofibrous substrates on the thin film composite forward osmosis membranes. *J. Environ. Chem. Eng.* **10**, 106958 (2022).
21 Zhao, Y. L., Lai, W. S., Wang, Y. N., Li, C. & Wang, R. Impact of pilot-scale PSF substrate surface and pore structural properties on tailoring seawater reverse osmosis membrane performance. *J. Membr. Sci.* **633**, 119395 (2021).
22 You, M. *et al.* Different roles of aqueous and organic additives in the morphology and performance of polyamide thin-film composite membranes. *Chem. Eng. Res. Des.* **165**, 1-11 (2021).
23 Shin, M. G., Seo, J. Y., Park, H., Park, Y. I. & Lee, J. H. Overcoming the permeability-selectivity trade-off of desalination membranes via controlled solvent activation. *J. Membr. Sci.* **620**, 118870 (2021).
24 Jung, K. H., Kim, H. J., Kim, M. H., Seo, H. & Lee, J. C. Superamphiphilic zwitterionic block copolymer surfactant-assisted fabrication of polyamide thin-film composite membrane with highly enhanced desalination performance. *J. Membr. Sci.* **618**, 118677 (2021).
25 Gonzales, R. R. *et al.* Aliphatic polyketone-based thin film composite membrane with mussel-inspired polydopamine intermediate layer for high performance osmotic power generation. *Desalination* **516**, 115222 (2021).
26 Cheng, X. J., Peng, Y., Li, S. X. & Su, B. W. Alginate hydrogel interlayer assisted interfacial polymerization for enhancing the separation performance of reverse osmosis membrane. *J. Membr. Sci.* **638**, 119680 (2021).
27 Jiang, C. *et al.* Ultrathin Film Composite Membranes Fabricated by Novel In Situ Free Interfacial Polymerization for Desalination. *ACS Appl. Mater. Interfaces* **12**, 25304-25315 (2020).
28 Wang, Z. Y., Liang, S. M., Jin, Y., Zhao, L. R. & Hu, L. J. Controlling structure and properties of polyamide nanofilms by varying amines diffusivity in organic phase. *J. Membr. Sci.* **574**, 1-9 (2019).
29 Shen, L. *et al.* High-performance thin-film composite polyamide membranes developed with green ultrasound-assisted interfacial polymerization. *J. Membr. Sci.* **570**, 112-119 (2019).
30 Xu, R. Z. *et al.* Influence of l-lysine on the permeation and antifouling performance of polyamide thin film composite reverse osmosis membranes. *Rsc Adv.* **8**, 25236-25247 (2018).
31 Li, R. P. *et al.* A thin film composite membrane supported by a hydrophilic poly(vinyl alcohol-co-ethylene) nanofiber membrane: Preparation, characterization, and application in nanofiltration. *J. Appl. Polym. Sci.* **135**, 46261 (2018).
32 Chowdhury, M. R., Steffes, J., Huey, B. D. & McCutcheon, J. R. 3D printed polyamide membranes for desalination. *Science* **361**, 682-685 (2018).
33 Zarshenas, K., Jiang, G. P., Zhang, J., Jauhar, M. A. & Chen, Z. W. Atomic scale

manipulation of sublayer with functional TiO2 nanofilm toward high-performance reverse osmosis membrane. *Desalination* **480**, 114342 (2020).
34 Park, S. J. & Lee, J. H. Fabrication of high-performance reverse osmosis membranes via dual-layer slot coating with tailoring interfacial adhesion. *J. Membr. Sci.* **614**, 118449 (2020).
35 Ng, Z. C., Lau, W. J., Kartohardjono, S. & Ismail, A. F. Comprehensive studies of membrane rinsing on the physicochemical properties and separation performance of TFC RO membranes. *Desalination* **491**, 114345 (2020).
36 Lim, Y. J., Lee, J., Bae, T. H., Torres, J. & Wang, R. Feasibility and performance of a thin-film composite seawater reverse osmosis membrane fabricated on a highly porous microstructured support. *J. Membr. Sci.* **611**, 118407 (2020).
37 Li, P. F. *et al.* Precise assembly of a zeolite imidazolate framework on polypropylene support for the fabrication of thin film nanocomposite reverse osmosis membrane. *J. Membr. Sci.* **612**, 118412 (2020).
38 Li, L. Q. *et al.* RO membrane fabricated via a facile modified heat-treating strategy for high-flux desalination. *J. Membr. Sci.* **614**, 118498 (2020).
39 Li, C. *et al.* Emerging sandwich-like reverse osmosis membrane with interfacial assembled covalent organic frameworks interlayer for highly-efficient desalination. *J. Membr. Sci.* **604**, 118065 (2020).
40 Shin, M. G. *et al.* Facile performance enhancement of reverse osmosis membranes via solvent activation with benzyl alcohol. *J. Membr. Sci.* **578**, 220-229 (2019).
41 Park, S. J. *et al.* Fabrication of polyamide thin film composite reverse osmosis membranes via support-free interfacial polymerization. *J. Membr. Sci.* **526**, 52-59 (2017).
42 Khorshidi, B., Thundat, T., Pernitsky, D. & Sadrzadeh, M. A parametric study on the synergistic impacts of chemical additives on permeation properties of thin film composite polyamide membrane. *J. Membr. Sci.* **535**, 248-257 (2017).
43 Zhang, J. L., Qin, Z. P., Yang, L. B., Guo, H. X. & Han, S. Y. Activation promoted ionic liquid modification of reverse osmosis membrane towards enhanced permeability for desalination. *J. Taiwan Inst. Chem. Eng.* **80**, 25-33 (2017).
44 Xu, J., Yan, H., Zhang, Y., Pan, G. Y. & Liu, Y. Q. The morphology of fully-aromatic polyamide separation layer and its relationship with separation performance of TFC membranes. *J. Membr. Sci.* **541**, 174-188 (2017).
45 Shi, M. Q., Wang, Z., Zhao, S., Wang, J. X. & Wang, S. C. A support surface pore structure re-construction method to enhance the flux of TFC RO membrane. *J. Membr. Sci.* **541**, 39-52 (2017).
46 Park, H. M., Jee, K. Y. & Lee, Y. T. Preparation and characterization of a thin-film composite reverse osmosis membrane using a polysulfone membrane including metal-organic frameworks. *J. Membr. Sci.* **541**, 510-518 (2017).
47 Lee, T. H. *et al.* Highly porous carbon nanotube/polysulfone nanocomposite supports for high-flux polyamide reverse osmosis membranes. *J. Membr. Sci.* **539**, 441-450 (2017).
48 Choi, W. *et al.* Thin film composite reverse osmosis membranes prepared via layered interfacial polymerization. *J. Membr. Sci.* **527**, 121-128 (2017).

49 Zhang, Y. *et al.* Highly improved permeation property of thin-film-composite polyamide membrane for water desalination. *J. Polym. Res.* **24**, 5 (2016).
50 Khorshidi, B., Thundat, T., Fleck, B. A. & Sadrzadeh, M. A Novel Approach Toward Fabrication of High Performance Thin Film Composite Polyamide Membranes. *Sci. Rep.* **6**, 22069 (2016).
51 Badalov, S. & Arnusch, C. J. Ink-jet printing assisted fabrication of thin film composite membranes. *J. Membr. Sci.* **515**, 79-85 (2016).
52 Duan, M. R., Wang, Z., Xu, J., Wang, J. X. & Wang, S. C. Influence of hexamethyl phosphoramide on polyamide composite reverse osmosis membrane performance. *Sep. Purif. Technol.* **75**, 145-155 (2010).
53 Li, Y. *et al.* Tailoring the Polyamide Active Layer of Thin-Film Composite Forward Osmosis Membranes with Combined Cosolvents during Interfacial Polymerization. *Ind. Eng. Chem. Res.* **59**, 8230-8242 (2020).
54 Xue, Y.-R. *et al.* Polyamide nanofilms synthesized by a sequential process of blade coating-spraying-interfacial polymerization toward reverse osmosis. *Sep. Purif. Technol.* **310**, 123122 (2023).
55 Ma, Z.-Y. *et al.* Demystifying viscous isoalkanes as the organic solvent in interfacial polymerization for manufacturing desalination membranes. *Desalination* **545**, 116166 (2023).
56 Rho, H. *et al.* Facile Surface Modification of Polyamide Membranes Using UV-Photooxidation Improves Permeability and Reduces Natural Organic Matter Fouling. *Environ. Sci. Technol.* **55**, 6984-6994 (2021).
57 Zhai, X. F., Meng, J. Q., Li, R., Ni, L. & Zhang, Y. F. Hypochlorite treatment on thin film composite RO membrane to improve boron removal performance. *Desalination* **274**, 136-143 (2011).
58 Park, S.-J. *et al.* Fabrication of polyamide thin film composite reverse osmosis membranes via support-free interfacial polymerization. *J. Membr. Sci.* **526**, 52-59 (2017).
59 Park, S.-H. *et al.* Polyethylene-supported high performance reverse osmosis membranes with enhanced mechanical and chemical durability. *Desalination* **436**, 28-38 (2018).
60 Tang, C. Y. Y., Kwon, Y. N. & Leckie, J. O. Effect of membrane chemistry and coating layer on physiochemical properties of thin film composite polyamide RO and NF membranes II. Membrane physiochemical properties and their dependence on polyamide and coating layers. *Desalination* **242**, 168-182 (2009).
61 Tang, C., Kwon, Y. & Leckie, J. Probing the nano- and micro-scales of reverse osmosis membranes—A comprehensive characterization of physiochemical properties of uncoated and coated membranes by XPS, TEM, ATR-FTIR, and streaming potential measurements. *J. Membr. Sci.* **287**, 146-156 (2007).
62 Liu, Z. X. *et al.* Thin-film composite nanofiltration membranes with poly (amidoxime) as organic interlayer for effective desalination. *J. Environ. Chem. Eng.* **10**, 107015 (2022).
63 Lu, Y. *et al.* Two-dimensional fractal nanocrystals templating for substantial performance enhancement of polyamide nanofiltration membrane. *Proc. Natl. Acad. Sci. U. S. A.* **118**, 118 (2021).

64 Wu, Q. Y. *et al.* Preparation and characterization of CeO2@high silica ZSM-5 inorganic-organic hybrid polyamide nanofiltration membrane. *J. Membr. Sci.* **641**, 119887 (2022).
65 Qin, Y. *et al.* Preparation of the interfacial enhanced PA/APVC nanofiltration membrane based on the in-situ amination of substrate membrane. *Sep. Purif. Technol.* **280**, 119964 (2022).
66 Zhao, B. *et al.* Enhanced water permeance of a polyamide thin-film composite nanofiltration membrane with a metal-organic framework interlayer. *J. Membr. Sci.* **625**, 119154 (2021).
67 Zhang, T. H., Li, P. Y., Ding, S. P. & Wang, X. F. High permeability composite nanofiltration membrane assisted by introducing TpPa covalent organic frameworks interlayer with nanorods for desalination and NaCl/dye separation. *Sep. Purif. Technol.* **270**, 118802 (2021).
68 Zhang, R. L. *et al.* Polyamide Nanofiltration Membranes from Surfactant-Assembly Regulated Interfacial Polymerization: The Effect of Alkyl Chain. *Macromol. Chem. Phys.* **222**, 2100222 (2021).
69 Yang, S. M. *et al.* Interfacial polymerized polyamide nanofiltration membrane by demulsification of hexane-in-water droplets through hydrophobic PTFE membrane: Membrane performance and formation mechanism. *Sep. Purif. Technol.* **275**, 119227 (2021).
70 Wu, J. H. *et al.* Facile preparation of polyvinylidene fluoride substrate supported thin film composite polyamide nanofiltration: Effect of substrate pore size. *J. Membr. Sci.* **638**, 119699 (2021).
71 Tian, J. Y. *et al.* Direct generation of an ultrathin (8.5 nm) polyamide film with ultrahigh water permeance via in-situ interfacial polymerization on commercial substrate membrane. *J. Membr. Sci.* **634**, 119450 (2021).
72 Tian, B. Z. *et al.* Nanofiltration membrane combining environmental-friendly polycarboxylic interlayer prepared from catechol for enhanced desalination performance. *Desalination* **512**, 115118 (2021).
73 Song, Q. Q. *et al.* Mechanism insights into the role of the support mineralization layer toward ultrathin polyamide nanofilms for ultrafast molecular separation. *J. Mater. Chem. A* **9**, 26159-26171 (2021).
74 Seah, M. Q., Khoo, Y. S., Lau, W. J., Goh, P. S. & Ismail, A. F. New Concept of Thin-Film Composite Nanofiltration Membrane Fabrication Using a Mist-Based Interfacial Polymerization Technique. *Ind. Eng. Chem. Res.* **60**, 9167-9178 (2021).
75 Ma, Z.-Y., Xue, Y.-R. & Xu, Z.-K. Alginate Hydrogel Assisted Controllable Interfacial Polymerization for High-Performance Nanofiltration Membranes. *Membranes* **11**, 435 (2021).
76 Lv, Y., Xia, J. J., Yang, Y., Chen, Y. Z. & Liu, T. X. Thin-film composite membranes with mineralized nanofiber supports for highly efficient nanofiltration. *Compos. Commun.* **24**, 100695 (2021).
77 Liu, Z. X., Wang, T., Wang, D. M. & Mi, Z. M. Regulating the morphology of nanofiltration membrane by thermally induced inorganic salt crystals for efficient water purification. *J. Membr. Sci.* **617**, 118645 (2021).

78 Liu, K. *et al.* Highly Permeable Polyamide Nanofiltration Membrane Mediated by an Upscalable Wet-Laid EVOH Nanofibrous Scaffold. *ACS Appl. Mater. Interfaces* **13**, 23142-23152 (2021).
79 Lan, H. L. *et al.* Construction of a gelatin scaffold with water channels for preparing a high performance nanofiltration membrane. *Sep. Purif. Technol.* **264**, 118391 (2021).
80 Jiang, Y. X. *et al.* Two dimensional COFs as ultra-thin interlayer to build TFN hollow fiber nanofiltration membrane for desalination and heavy metal wastewater treatment. *J. Membr. Sci.* **635**, 119523 (2021).
81 Choi, O., Peck, D. H. & Park, C. H. High-performance nanofiltration of outer-selective thin-film composite hollow-fiber membranes via continuous interfacial polymerization. *J. Ind. Eng. Chem.* **103**, 373-380 (2021).
82 Bian, X. Y., Zhang, Y. P., Gong, L., Zhu, Y. Z. & Jin, J. Calcium Ion Coordinated Polyamide Nanofiltration Membrane for Ultrahigh Perm-selectivity Desalination. *Chem. Res. Chin. Univ.* **37**, 1101-1109 (2021).
83 Zhang, X. *et al.* Nanofiltration membranes with hydrophobic microfiltration substrates for robust structure stability and high water permeation flux. *J. Membr. Sci.* **593**, 117444 (2020).
84 Yuan, S. S. *et al.* Hydrogel assisted interfacial polymerization for advanced nanofiltration membranes. *J. Mater. Chem. A* **8**, 3238-3245 (2020).
85 Yang, X. Monitoring the Interfacial Polymerization of Piperazine and Trimesoyl Chloride with Hydrophilic Interlayer or Macromolecular Additive by In Situ FT-IR Spectroscopy. *Membranes* **10**, 12 (2020).
86 Tian, J. Y., Chang, H. L., Gao, S. S. & Zhang, R. J. How to fabricate a negatively charged NF membrane for heavy metal removal via the interfacial polymerization between PIP and TMC? *Desalination* **491**, 114499 (2020).
87 Sun, H. X. *et al.* Fabrication of thin-film composite polyamide nanofiltration membrane based on polyphenol intermediate layer with enhanced desalination performance. *Desalination* **488**, 114525 (2020).
88 Hu, P. *et al.* Application of diazonium-induced anchoring process on ultrafiltration substrate for the fabrication of nanofiltration membrane with enhanced desalination performance. *Desalination* **496**, 114340 (2020).
89 He, M. B. *et al.* Performance improvement for thin-film composite nanofiltration membranes prepared on PSf/PSf-g-PEG blended substrates. *Sep. Purif. Technol.* **230**, 115855 (2020).
90 Han, S. Q. *et al.* Root-like polyamide membranes with fast water transport for high-performance nanofiltration. *J. Mater. Chem. A* **8**, 25028 (2020).
91 Gao, S. J. *et al.* Ultrathin Polyamide Nanofiltration Membrane Fabricated on Brush-Painted Single-Walled Carbon Nanotube Network Support for Ion Sieving. *ACS Nano* **13**, 5278-5290 (2019).
92 Chen, M. X., Xiao, C. F., Wang, C., Liu, H. L. & Huang, N. Z. Preparation and characterization of a novel thermally stable thin film composite nanofiltration membrane with poly (m-phenyleneisophthalamide) (PMIA) substrate. *J. Membr. Sci.* **550**, 36-44 (2018).
93 Yang, X., Du, Y., Zhang, X., He, A. & Xu, Z.-K. Nanofiltration Membrane with a

Mussel-Inspired Interlayer for Improved Permeation Performance. *Langmuir* **33**, 2318-2324 (2017).
94 Wang, X., Xiao, Q., Wu, C., Li, P. & Xia, S. Fabrication of nanofiltration membrane on MoS2 modified PVDF substrate for excellent permeability, salt rejection, and structural stability. *Chem. Eng. J.* **416**, 129154 (2021).
95 Zhu, C.-Y. *et al.* Polyamide nanofilms with linearly-tunable thickness for high performance nanofiltration. *J. Membr. Sci.* **627**, 119142 (2021).
96 Zhu, C.-Y. *et al.* Vacuum-assisted diamine monomer distribution for synthesizing polyamide composite membranes by interfacial polymerization. *J. Membr. Sci.* **616**, 118557 (2020).
97 Wu, M.-B. *et al.* Thin film composite membranes combining carbon nanotube intermediate layer and microfiltration support for high nanofiltration performances. *J. Membr. Sci.* **515**, 238-244 (2016).
98 Wang, J.-J., Yang, H.-C., Wu, M.-B., Zhang, X. & Xu, Z.-K. Nanofiltration membranes with cellulose nanocrystals as an interlayer for unprecedented performance. *J. Mater. Chem. A* **5**, 16289-16295 (2017).
99 Zhu, X. *et al.* Stainless steel mesh supported thin-film composite nanofiltration membranes for enhanced permeability and regeneration potential. *J. Membr. Sci.* **618**, 118738 (2021).
100 Zhu, X. *et al.* In-situ covalently bonded supramolecular-based protective layer for improving chlorine resistance of thin-film composite nanofiltration membranes. *Desalination* **474**, 114197 (2020).
101 Ritt, C. L. *et al.* Ionization behavior of nanoporous polyamide membranes. *Proc. Natl. Acad. Sci. U. S. A.* **117**, 30191-30200 (2020).
102 Rho, H., Chon, K. & Cho, J. Surface charge characterization of nanofiltration membranes by potentiometric titrations and electrophoresis: Functionality vs. zeta potential. *Desalination* **427**, 19-26 (2018).
103 Coronell, O., Marinas, B. J., Zhang, X. J. & Cahill, D. G. Quantification of functional groups and modeling of their ionization behavior in the active layer of FT30 reverse osmosis membrane. *Environ. Sci. Technol.* **42**, 5260-5266 (2008).
104 Perry, L. A. & Coronell, O. Reliable, bench-top measurements of charge density in the active layers of thin-film composite and nanocomposite membranes using quartz crystal microbalance technology. *J. Membr. Sci.* **429**, 23-33 (2013).
105 Baer, D. R. *et al.* Practical guides for x-ray photoelectron spectroscopy: First steps in planning, conducting, and reporting XPS measurements. *J. Vac. Sci. Technol. A: Vacuum, Surfaces, and Films* **37**, 031401 (2019).
106 Stevie, F. A., Garcia, R., Shallenberger, J., Newman, J. G. & Donley, C. L. Sample handling, preparation and mounting for XPS and other surface analytical techniques. *J. Vac. Sci. Technol. A: Vacuum, Surfaces, and Films* **38**, 063202 (2020).
107 Baer, D. R., Engelhard, M. H. & Lea, A. S. Introduction to Surface Science Spectra data on electron and x-ray damage: Sample degradation during XPS and AES measurements. *Surf. Sci. Spectra* **10**, 47-56 (2003).
108 Stevie, F. A. & Donley, C. L. Introduction to x-ray photoelectron spectroscopy. *J. Vac. Sci. Technol. A: Vacuum, Surfaces, and Films* **38**, 063204 (2020).

109 Ariza, M. J., Prádanos, P., Rico, R., Rodríguez-Castellón, E. & Benavente, J. X-ray action on polymeric membrane surfaces: a chemical and morphological characterization. *Surf. Interface Anal.* **35**, 360-368 (2003).
110 Raaijmakers, M. J. T. & Benes, N. E. Current trends in interfacial polymerization chemistry. *Prog. Polym. Sci.* **63**, 86-142 (2016).
111 Farahbakhsh, J., Vatanpour, V., Khoshnam, M. & Zargar, M. Recent advancements in the application of new monomers and membrane modification techniques for the fabrication of thin film composite membranes: A review. *React. Funct. Polym.* **166**, 105015 (2021).
112 Zhang, C., Wu, B. H., Ma, M. Q., Wang, Z. & Xu, Z. K. Ultrathin metal/covalent-organic framework membranes towards ultimate separation. *Chem. Soc. Rev.* **48**, 3811-3841 (2019).
113 Yang, Z., Guo, H. & Tang, C. Y. The upper bound of thin-film composite (TFC) polyamide membranes for desalination. *J. Membr. Sci.* **590**, 117297 (2019).
114 Habib, S. & Weinman, S. T. A review on the synthesis of fully aromatic polyamide reverse osmosis membranes. *Desalination* **502**, 114939 (2021).
115 Li, Y., Wong, E., Mai, Z. H. & Van der Bruggen, B. Fabrication of composite polyamide/Kevlar aramid nanofiber nanofiltration membranes with high permselectivity in water desalination. *J. Membr. Sci.* **592**, 117396 (2019).
116 Zhu, Y. Z. *et al.* Single-Walled Carbon Nanotube Film Supported Nanofiltration Membrane with a Nearly 10 nm Thick Polyamide Selective Layer for High-Flux and High-Rejection Desalination. *Small* **12**, 5034-5041 (2016).
117 Zhao, Y. Y., Tong, X. & Chen, Y. S. Fit-for-Purpose Design of Nanofiltration Membranes for Simultaneous Nutrient Recovery and Micropollutant Removal. *Environ. Sci. Technol.* **55**, 3352-3361 (2021).
118 Trivedi, J. S., Bhalani, D. V., Bhadu, G. R. & Jewrajka, S. K. Multifunctional amines enable the formation of polyamide nanofilm composite ultrafiltration and nanofiltration membranes with modulated charge and performance. *J. Mater. Chem. A* **6**, 20242-20253 (2018).
119 Wu, M.-B. *et al.* Positively-charged nanofiltration membranes constructed via gas/liquid interfacial polymerization for Mg2+/Li+ separation. *J. Membr. Sci.* **644**, 119942 (2022).
120 Han, R. L. Formation and characterization of (melamine-TMC) based thin film composite NF membranes for improved thermal and chlorine resistances. *J. Membr. Sci.* **425**, 176-181 (2013).
121 Yu, S. C., Liu, M. H., Lu, Z. H., Zhou, Y. & Gao, C. J. Aromatic-cycloaliphatic polyamide thin-film composite membrane with improved chlorine resistance prepared from m-phenylenediamine-4-methyl and cyclohexane-1,3,5-tricarbonyl chloride. *J. Membr. Sci.* **344**, 155-164 (2009).
122 Ounifi, I. *et al.* Synthesis of a Thin-Film Polyamide-Cellulose Acetate Membrane: Effect of Monomers and Porosity on Nano-Filtration Performance. *J. Nat. Fibers*, 1-18 (2021).
123 Ali, Z. *et al.* Gas separation and water desalination performance of defect-free interfacially polymerized para-linked polyamide thin-film composite membranes. *J. Membr. Sci.* **618**, 118572 (2021).

124 Wang, T. Y., Dai, L., Zhang, Q. F., Li, A. & Zhang, S. B. Effects of acyl chloride monomer functionality on the properties of polyamide reverse osmosis (RO) membrane. *J. Membr. Sci.* **440**, 48-57 (2013).

125 Wang, T. Y., Yang, Y. Q., Zheng, J. F., Zhang, Q. F. & Zhang, S. B. A novel highly permeable positively charged nanofiltration membrane based on a nanoporous hyper-crosslinked polyamide barrier layer. *J. Membr. Sci.* **448**, 180-189 (2013).

126 Saha, N. K. & Joshi, S. V. Performance evaluation of thin film composite polyamide nanofiltration membrane with variation in monomer type. *J. Membr. Sci.* **342**, 60-69 (2009).

127 Li, L., Zhang, S. B., Zhang, X. S. & Zheng, G. D. Polyamide thin film composite membranes prepared from isomeric biphenyl tetraacyl chloride and m-phenylenediamine. *J. Membr. Sci.* **315**, 20-27 (2008).

128 Liu, Y. *et al.* Formation and structural evolution of biphenyl polyamide thin film on hollow fiber membrane during interfacial polymerization. *J. Membr. Sci.* **373**, 98-106 (2011).

129 Li, L., Zhang, S. B. & Zhang, X. S. Preparation and characterization of poly(piperazineamide) composite nanofiltration membrane by interfacial polymerization of 3,3 ',5,5 '-biphenyl tetraacyl chloride and piperazine. *J. Membr. Sci.* **335**, 133-139 (2009).

130 Tang, B. B., Huo, Z. B. & Wu, P. Y. Study on a novel polyester composite nanofiltration membrane by interfacial polymerization of triethanolamine (TEOA) and trimesoyl chloride (TMC) I. Preparation, characterization and nanofiltration properties test of membrane. *J. Membr. Sci.* **320**, 198-205 (2008).

131 Kim, S., Lim, H., Lee, J. & Choi, H. C. Synthesis of a Scalable Two-Dimensional Covalent Organic Framework by the Photon-Assisted (mine Condensation Reaction on the Water Surface. *Langmuir* **34**, 8731-8738 (2018).

132 Feldblyum, J. I. *et al.* Few-layer, large-area, 2D covalent organic framework semiconductor thin films. *Chem. Commun.* **51**, 13894-13897 (2015).

133 Gadwal, I. *et al.* Synthesis of Sub-10 nm Two-Dimensional Covalent Organic Thin Film with Sharp Molecular Sieving Nanofiltration. *ACS Appl. Mater. Interfaces* **10**, 12295-12299 (2018).

134 Shinde, D. B. *et al.* Crystalline 2D Covalent Organic Framework Membranes for High-Flux Organic Solvent Nanofiltration. *J. Am. Chem. Soc.* **140**, 14342-14349 (2018).

135 Abu Seman, M. N., Khayet, M. & Hilal, N. Nanofiltration thin-film composite polyester polyethersulfone-based membranes prepared by interfacial polymerization. *J. Membr. Sci.* **348**, 109-116 (2010).

136 Tatiya, P. D., Hedaoo, R. K., Mahulikar, P. P. & Gite, V. V. Novel Polyurea Microcapsules Using Dendritic Functional Monomer: Synthesis, Characterization, and Its Use in Self-healing and Anticorrosive Polyurethane Coatings. *Ind. Eng. Chem. Res.* **52**, 1562-1570 (2013).

137 Lee, K. P., Zheng, J. M., Bargeman, G., Kemperman, A. J. B. & Benes, N. E. pH stable thin film composite polyamine nanofiltration membranes by interfacial polymerisation. *J. Membr. Sci.* **478**, 75-84 (2015).
